# The Emirates Mars Mission

**DOI:** 10.1007/s11214-021-00868-x

**Published:** 2022-02-10

**Authors:** H. E. S. Amiri, D. Brain, O. Sharaf, P. Withnell, M. McGrath, M. Alloghani, M. Al Awadhi, S. Al Dhafri, O. Al Hamadi, H. Al Matroushi, Z. Al Shamsi, O. Al Shehhi, M. Chaffin, J. Deighan, C. Edwards, N. Ferrington, B. Harter, G. Holsclaw, M. Kelly, D. Kubitschek, B. Landin, R. Lillis, M. Packard, J. Parker, E. Pilinski, B. Pramman, H. Reed, S. Ryan, C. Sanders, M. Smith, C. Tomso, R. Wrigley, H. Al Mazmi, N. Al Mheiri, M. Al Shamsi, E. Al Tunaiji, K. Badri, P. Christensen, S. England, M. Fillingim, F. Forget, S. Jain, B. M. Jakosky, A. Jones, F. Lootah, J. G. Luhmann, M. Osterloo, M. Wolff, M. Yousuf

**Affiliations:** 1 UAE Ministry of Industry and Advanced Technology, Abu Dhabi, United Arab Emirates; 2 Laboratory for Atmospheric and Space Physics, University of Colorado, Boulder, USA; 3 Mohammed Bin Rashid Space Centre, Dubai, United Arab Emirates; 4 Northern Arizona University, Flagstaff, AZ USA; 5 Advanced Space, Boulder, CO USA; 6 NASA Goddard Space Flight Center, Greenbelt, MD USA; 7 Space Sciences Lab, University of California, Berkeley, USA; 8 Laboratoire de Météorologie Dynamique, Paris, France; 9 UAE Space Agency, Abu Dhabi, United Arab Emirates; 10 Space Science International, Boulder, CO USA; 11 Arizona State University, Tempe, AZ USA; 12 Virgina Tech University, Blacksburg, VA USA

**Keywords:** Mars, Atmosphere, EMM, Hope

## Abstract

The Emirates Mars Mission (EMM) was launched to Mars in the summer of 2020, and is the first interplanetary spacecraft mission undertaken by the United Arab Emirates (UAE). The mission has multiple programmatic and scientific objectives, including the return of scientifically useful information about Mars. Three science instruments on the mission’s Hope Probe will make global remote sensing measurements of the Martian atmosphere from a large low-inclination orbit that will advance our understanding of atmospheric variability on daily and seasonal timescales, as well as vertical atmospheric transport and escape. The mission was conceived and developed rapidly starting in 2014, and had aggressive schedule and cost constraints that drove the design and implementation of a new spacecraft bus. A team of Emirati and American engineers worked across two continents to complete a fully functional and tested spacecraft and bring it to the launchpad in the middle of a global pandemic. EMM is being operated from the UAE and the United States (U.S.), and will make its data freely available.

## Introduction

In the early morning hours of July 20, 2020 (July 19, UTC), the Emirates Mars Mission (EMM) was launched from the Tanegashima Space Center in Japan in the midst of a global pandemic. The mission, consisting of a scientific probe called Hope (Al Amal, in Arabic), is the first interplanetary spacecraft developed by the United Arab Emirates (UAE). Led by the Mohammed bin Rashid Space Centre (MBRSC) in the UAE, the mission was jointly conceived and developed over a six year period by MBRSC and its Knowledge Transfer partners at the University of Colorado Boulder, Arizona State University, and the University of California, Berkeley. Hope arrived at Mars on 9 February 2021.

The mission will study the Martian atmosphere for one Martian year using three scientific instruments that will make measurements at infrared, visible, and ultraviolet wavelengths: the Emirates Mars InfraRed Spectrometer (EMIRS), the Emirates eXploration Imager (EXI), and the Emirates Mars Ultraviolet Spectrometer (EMUS). The unprecedented large orbit and low orbital inclination of the spacecraft will allow global views of the Martian lower and upper atmosphere at all local times, enabling new understanding of the Martian atmospheric transport (both horizontal and vertical) on diurnal and seasonal timescales.

The Laboratory for Atmospheric and Space Physics (LASP) at University of Colorado, Boulder is the primary knowledge transfer partner for mission design, spacecraft development, EXI/EMIRS instrument development, testing, science team and science apprenticeship, and operations. Arizona State University is the knowledge transfer partner for the EMIRS instrument development and science team and science apprenticeship. The University of California, Berkeley Space Sciences Lab (SSL) is the knowledge transfer partner for science team and science apprenticeship and the EMUS detectors.

Development on EMM began in 2014, and followed a novel timeline and approach that required planning, review, implementation, and testing that occurred on two continents, with team members from both UAE and the U.S. spending long periods living and working abroad. Activities in the final months before launch were conducted as the Corona Virus Disease 2019 (COVID-19) pandemic struck countries around the world, including the UAE (where spacecraft testing occurred), the U.S., and Japan (where the launch site was located). Spacecraft operations continue with the main operations center located at MBRSC in Dubai, UAE and the backup at LASP in Boulder, Colorado.

This paper describes the EMM mission programmatic and scientific goals, scientific approach, mission plan, mission team, spacecraft, testing, and operations and data availability. Companion papers in this issue describe the science closure plan for the mission and the three scientific instruments.

## Mission Objectives

### Programmatic Objectives

EMM was conceived to satisfy a number of programmatic objectives for the UAE. These objectives are centered around the themes of capacity-building as part of transitioning the national economy to be based on science and technology, and building a national and regional identity as a space-faring nation. Program objectives set by the UAE government include:

- Complete the insertion of a Mars orbiter by the UAE’s 50th anniversary in 2021
- Contribute to development of the science and technology sector in the UAE
- Develop scientific capabilities in the UAE
- Increase the UAE’s contribution to the scientific community

The following objectives were derived from the above goals:

- Train and prepare Emirati scientists to do significant science work in the field of space exploration
- Train and prepare Emirati engineers to develop outer space exploration systems and instruments in the UAE
- Build the necessary infrastructure to create a sustainable outer space exploration program in the UAE
- Establish partnership with international entities in the field of outer space exploration
- Establish, improve and further develop the engineering and scientific programs in the academic sector
- Transfer knowledge to the different sectors in UAE (e.g. via spin-offs and spillover effects)

To satisfy these objectives, the following requirements were established to develop the EMM mission concept:

- The mission should be unique, and should aim for novel and significant discoveries
- The mission should have significant contributions to the ongoing work of the global space science community, and should be of great value to humanity
- The mission should help build a sustainable outer space exploration program in the UAE
- The mission should include valuable contribution from Emirati engineers and scientists
- Some of the system development activities should take place in the UAE

These requirements guided the mission concept, development, and implementation.

### Science Objectives

EMM has three science objectives focused on revealing the state and variability of the Martian atmosphere. These objectives, addressing the lower atmosphere, connections between the lower and upper atmosphere, and the upper atmosphere, guide the science investigations that EMM will complete, as well as requirements on the instruments and mission. Each objective is presented briefly below, and discussed more thoroughly in the companion paper by Almatroushi et al. (2021).

#### Objective A: Characterize the State of the Martian Lower Atmosphere on Global Scales and Its Geographic, Diurnal, and Seasonal Variability

Although considerable progress has been made toward understanding Mars atmosphere and climate by current and previous spacecraft over the past few decades (see the reviews by Smith et al. 2017; Kahre et al. 2017; Clancy et al. 2017; Montmessin et al. 2017; Wolff et al. 2017, and references therein), there are still significant gaps in our knowledge that limit our ability to understand the links between solar forcing and lower atmosphere variations. A large majority of incoming solar radiation is absorbed, reflected, or scattered by gases and aerosols in the lower atmosphere (which includes altitudes below 50 km) or the surface (e.g. Wolff et al. 2017). Therefore, characterizing the lower atmosphere state and how it varies is a crucial first step toward understanding energy balance and the physical processes that control transport and escape processes. The suite of science instruments on EMM has been carefully chosen to acquire the observations needed to retrieve the key quantities for characterizing the lower atmospheric state, which include surface and atmospheric temperature, column optical depth of dust and water ice aerosol, and column abundance of water vapor and ozone.

**Fig. 1:**
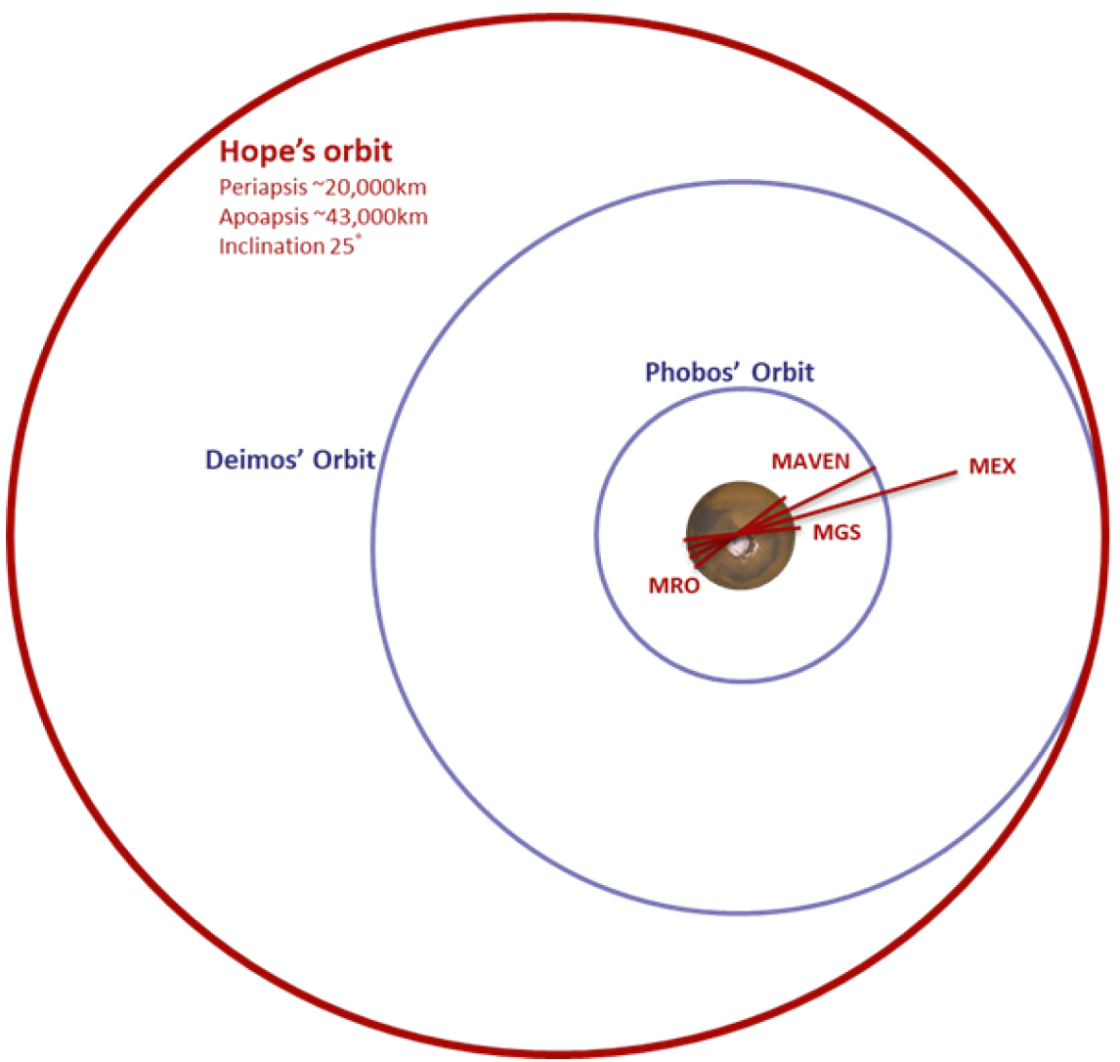
Cartoon of the size and inclination of Hope’s orbit relative to the orbit size of the Martian moons Phobos and Deimos, as well as several active Mars missions at the time of launch of EMM

Two key gaps in our current understanding are the diurnal variation of atmospheric state quantities and a global-scale view over short time periods. In the lower atmosphere the diurnal cycle drives atmospheric circulation and solar tides, surface-atmosphere interactions including dust lifting and water exchange, and the diurnal variation of the water cycle through condensation processes. A global synoptic view of Mars will permit characterization of the time evolution of dust storms, cloud formation, and their associated radiative effects. The unique orbit of EMM (Fig. 1) has been designed to enable atmospheric state quantities to be retrieved globally for all local times in just 10 days (see Sect. 3.3 for details). These novel observations from EMM will greatly help in understanding these important drivers for energy exchange between the lower and upper atmosphere.

#### Objective B: Correlate Rates of Thermal and Photochemical Atmospheric Escape with Conditions in the Collisional Martian Atmosphere

Unlike EMM’s Objectives A and C which each focus on understanding a region of the atmosphere independently (e.g. lower atmosphere, exosphere), Objective B targets the connections between regions. Many recent results (described in detail below) suggest that the lower and upper atmosphere of Mars are intimately linked in ways that were not fully expected. Objective B focuses on understanding and constraining these connections through correlation analysis and related modeling studies.

In quiescent times, large-amplitude short-term variability in temperature measurements indicates that wave and tidal activity plays a strong role in mediating the connection between the lower and upper atmosphere, with potential for significant impacts on atmospheric escape and evolution (Jain et al. 2020; Bougher et al. 2017). Observations of global-scale oscillations in density, pressure, and temperature have revealed non-migrating tides driven by diurnal solar forcing, with periods which are harmonics of the solar day (Lo et al. 2015; England et al. 2016; Schneider et al. 2020). These past observations have been significantly limited by a lack of consistent global-scale coverage; EMM will provide these observations, enabling wave and tidal analysis of both lower and upper atmospheric parameters.

Dust storms are known to have major impacts on the dynamics and composition of the upper atmosphere. Dynamically, large increases in the altitude and temperature of the thermosphere have been observed during dust activity (Jain et al. 2020), along with significant increases in turbulent wave activity (Wu et al. 2020; Liuzzi et al. 2020; Connour et al. 2020). Compositional differences can result from dust activity, with observed decreases in thermospheric oxygen concentration (Elrod et al. 2020).

Perhaps the most dramatic influence of the lower atmosphere on the upper is the increase in H concentration and escape rate induced by dust activity. While the exact mechanisms of the enhancement are not known, H escape is greatly enhanced with season (Chaffin et al. 2014; Clarke et al. 2014; Bhattacharyya et al. 2015; Halekas 2017), and H loss strongly responds to some regional dust storms (Chaffin et al. 2021). The enhancement is thought to be related to increased water concentrations at altitudes greater than 20 km, which are enabled by higher atmospheric temperatures resulting from dust heating (Maltagliati et al. 2011, 2013; Fedorova et al. 2018; Heavens et al. 2018; Aoki et al. 2019; Fedorova et al. 2020). Observations of H-bearing thermospheric species (Stone et al. 2020) as well as photochemical and dynamical modeling studies (Chaffin et al. 2017; Shaposhnikov et al. 2019; Neary et al. 2020) confirm the plausibility of this mechanism. Despite these prior observations, significant unanswered questions about dust-driven H escape remain, including whether H is enhanced by all dust activity or only certain kinds, whether the upper atmospheric response is restricted geographically depending on the lower atmospheric input, and how much H loss is attributable to dust rather than other drivers of escape.

By measuring lower atmospheric temperatures, the dust cycle, and the upper atmosphere simultaneously at all phase angles across the full Martian year, EMM will enable a more comprehensive understanding of their connection than ever before.

#### Objective C: Characterize the Spatial Structure and Variability of Key Constituents in the Martian Exosphere

The exosphere is the upper-most region of the Martian atmosphere, where inter-molecular collisions are infrequent and atoms escaping to space originate, making it critical for understanding the evolution of the planet’s atmosphere (Johnson et al. 2008). The bottom of the exosphere, termed the exobase, is defined as being where the density scale height is comparable to the molecular mean free path. This allows atmospheric particles to travel great distances on ballistic trajectories and even escape the planet if they possess sufficient velocity. The upper reaches of the exosphere are populated by the lightest and most energetic species, which form a diffuse corona of gravitationally bound and escaping gas that extends to great distances from the planet. The dominant species in the extended corona of Mars are thermal atomic H (Anderson and Hord 1971; Chaufray et al. 2008; Chaffin et al. 2015) and photochemically produced non-thermal atomic O generated primarily by dissociative recombination of O2+ in the ionosphere (e.g. McElroy 1972; Wallis 1978; Feldman et al. 2011; Deighan et al. 2015). There is some observational evidence for a non-thermal population of atomic H as well, which, though a minor contributor to coronal density, could contribute significantly to the escape rate of H (Bhattacharyya et al. 2015; Chaffin et al. 2018).

Though collisionless, the gas in the exosphere is highly influenced by the geographic variations in composition, temperature, and photochemistry in the collisional atmosphere from which it is ultimately sourced. In particular, the thermal atomic H is strongly sculpted by the diurnal and latitudinal circulation and temperature gradients (Chaufray et al. 2015) and the availability of H bearing species from the lower atmosphere (see Sect. 2.2.2 Objective B), while the production of non-thermal atomic O is modulated by the atmospheric solar ionization rate, ionospheric composition, and electron temperature (see review by Lillis et al. 2015, and references therein). A comprehensive study of the exosphere’s spatial structure and temporal variation thus requires geographic coverage of all local times and latitudes ranging from the equator to polar regions. The exosphere also contains significant radial structure, determined primarily by the energy distributions of the H and O atoms. This in turn depends on the exospheric neutral temperature for the thermal atomic H and the ion temperature and dissociative recombination branching ratios of various electronically excited states for the atomic O.

The EMM mission concept sub-divides the exosphere into three notional regions: (1) an “inner” region from 1.06-1.6 $R_{M}$ (Mars radii) where the corona is mostly gravitationally bound and remains strongly coupled to spatial variations in the exobase source region, (2) a “middle” region from 1.6-6.0 $R_{M}$ where the fraction of atoms that are gravitationally bound declines with altitude and the effects of local variations across the planet become blurred, and (3) an “upper” region extending above 6.0 RM where a large fraction of the atoms are gravitationally unbound and on their way to escaping from the planet. To characterize the global Martian exosphere, the EMUS instrument will measure the geographic and radial variations by observing the solar resonant fluorescence of the H I 102.6 nm, H I 121.6 nm, and O I 130.4 nm atomic transitions in these three regions, with temporal sampling that resolves seasonal variations. The ultraviolet brightness observations are converted to geophysical densities as part of the EMUS data processing, synthesizing multiple points of view around the planet to reconstruct the 3D structure of the corona. These observations will be compared with physics based models to solidify our understanding of how the exosphere is populated and the drivers of atmospheric escape at Mars.

## Scientific Approach

### Science Traceability

**Fig. 2:**
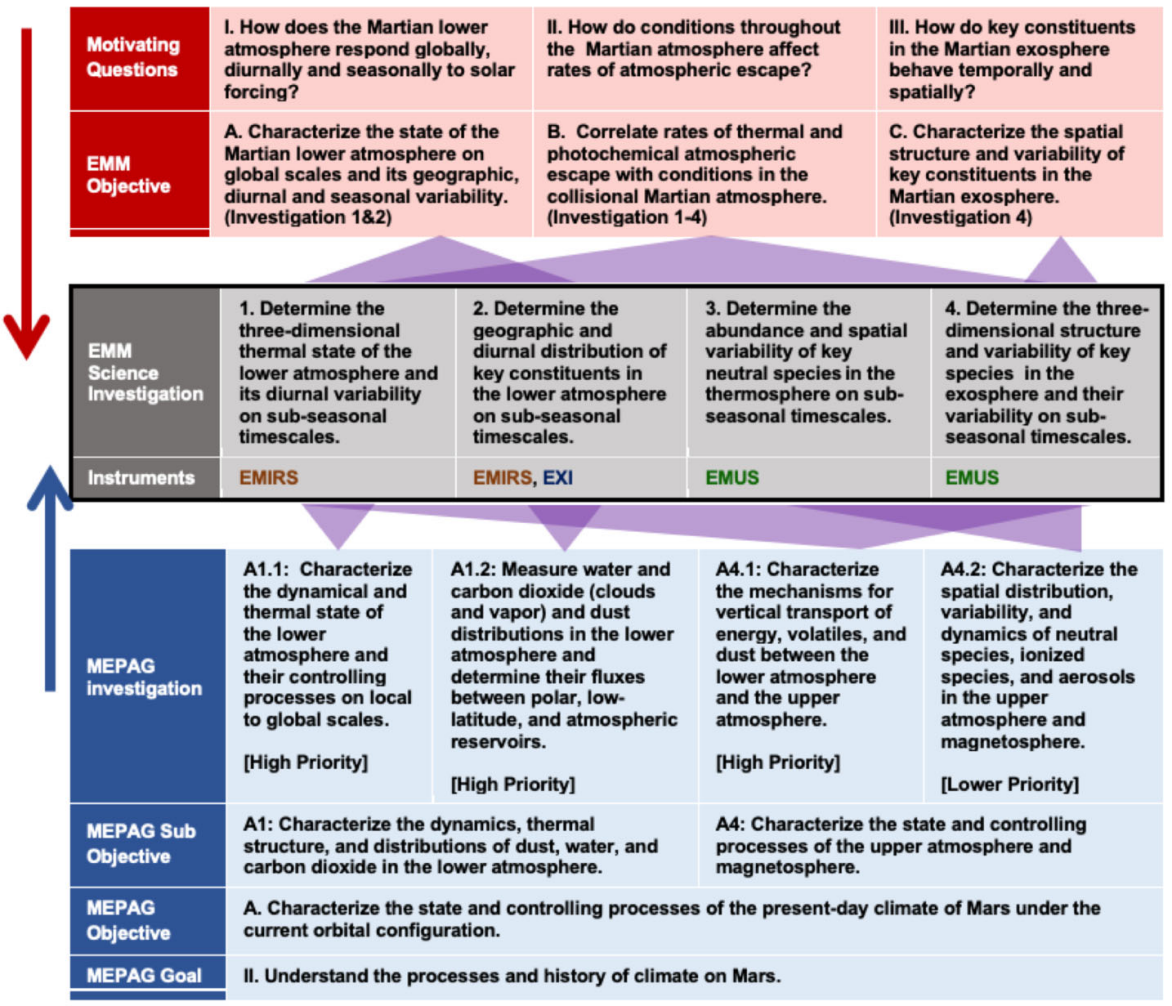
Traceability to EMM Investigations, measurements and instruments from Motivating Questions and EMM Science Objectives (upper half of figure) and from MEPAG Goals, Objectives and Investigations (lower half of figure)

To achieve the EMM Science Objectives described in Sect. 2.2, Hope must make accurate measurements of relevant quantities in the Martian atmosphere as a function of altitude, latitude, longitude, local time, and season. Figure 2 shows the traceability to EMM’s Science Investigations from two directions: downward from the EMM motivating Science Questions and Objectives and upward from the Mars Exploration Program Analysis Group (MEPAG) Goals and Objectives. The MEPAG Goals document (MEPAG 2020) is regularly updated to represent a consensus view of priorities for the scientific exploration of Mars, formulated by the scientific community in the U.S. and beyond.

**Fig. 3:**
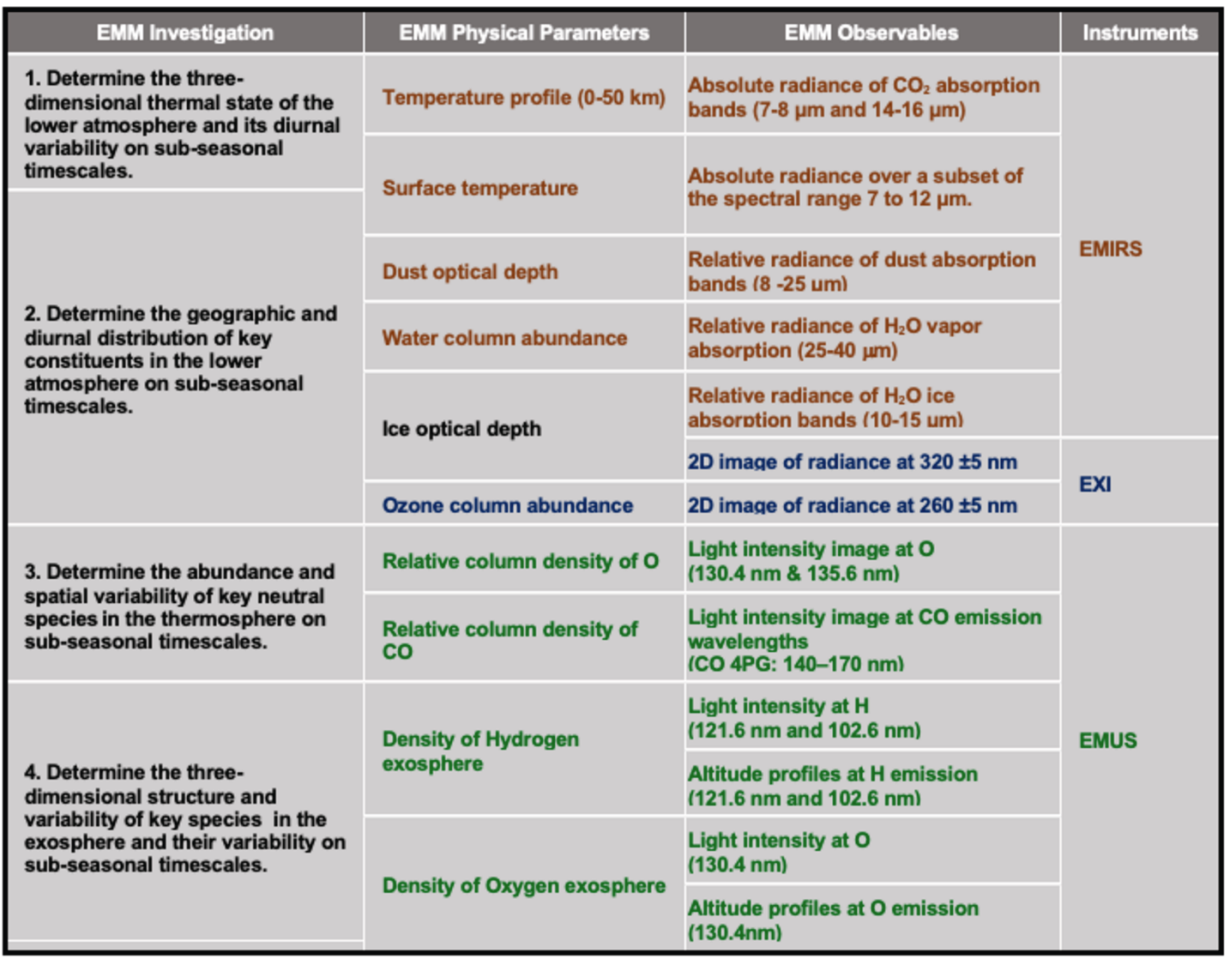
Traceability between EMM Science Investigations, the required physical parameters of the Mars climate system, the observable quantities necessary to determine those physical parameters, and finally the instruments that will measure the observables

The logical progression from science questions to specific requirements on the EMM instruments and flight system is captured in the project’s Science Traceability Matrix (STM). EMM’s three motivating *Science Questions* flow one-to-one to three *Mission Objectives* necessary to comprehensively address those questions. The Mission Objectives flow, in turn, to four *Science Investigations* (i.e. sets of measurements) necessary to achieve the defined objectives. The Science Investigations can be mapped to four recommended MEPAG Investigations, with three of them labeled as high priority as of 2020. The completion of each EMM Science Investigation requires the determination of certain physical parameters of the Mars climate system. Where applicable, the STM specifies the range over which, and accuracy with which, EMM must determine these parameters. The STM then describes the *Observable Quantities* from EMM’s orbit that are necessary to determine the required physical parameters. These are reflected and emitted energy at various wavelengths or over various bands in the ultraviolet, visible and thermal infrared, as shown in Fig. 3. The STM also contains the requirements placed on the measurements of those quantities: accuracy, range, resolution, cadence, measurement position and coverage of Mars with respect to geography, solar zenith angle, local time, or season. Lastly, the STM describes the *Functional Requirements* on both the instruments and the mission (e.g. orbit parameters) which are necessary to meet the measurement requirements for the observable quantities.

The full detail of this progression is captured in the STM for each investigation, but is outside the scope of this mission overview paper. Here, we demonstrate the form of the traceability with the example of Investigation 1, which is conducted with the EMIRS instrument.

Investigation 1 will “Determine the three-dimensional thermal state of the lower atmosphere and its diurnal variability on sub-seasonal timescales”. Atmospheric temperature is the physical parameter that must be determined, from the surface up to 50 km altitude. The required spatial resolution is <10 km in the vertical direction and <300 km at nadir in the horizontal direction. The required accuracy is ±2 °K at the surface and up to 25 km, ±4 °K from 25 to 40 km, and ±10 °K from 40 to 50 km.

To derive temperatures, the relevant observable quantity is thermal infrared radiance, which must be determined with an absolute radiometric accuracy of <1.5% and spectral resolution of <10 cm-1. Surface temperatures are derived from absolute radiance over a subset of the spectral range 7-12 μm, while temperature in the atmosphere is derived from the absolute radiance of $\mathrm{CO}_{2}$ absorption bands at 7-8 μm and 14-16 μm [Edwards et al., this issue].

To achieve sufficient coverage of the Martian day-night cycle, in any given span of 10 days EMIRS must measure these thermal radiances over >80% of Martian longitudes in at least 6 of 8 local time intervals for all latitudes equatorward of ±30° and at least 4 of 8 local time intervals for latitudes equatorward of ±50°. To achieve sufficient geographic coverage, EMIRS must measure the radiances over at least 80% of the geographic area of Mars (regardless of local time) every 72 hours. To achieve sufficient seasonal coverage, the above observations must be made in at least 20 of the 24 15° intervals of solar longitude ($\mathrm{L_{S}}$) over a full Martian year.

Finally, in order to make these measurements with the above required fidelity, the EMM mission must fulfill certain functional requirements. Measurements must be made from an orbital platform whose altitude is between 15,000 km and 43,000 km and whose orbital inclination is between 15° and 25°. This platform must be capable of slewing the EMIRS field of view (FOV) across the entire Martian disk with a 3-sigma accuracy of 1°or less.

A similar traceability from investigations to physical parameters to observable quantities and functional requirements exists for Investigations 2, 3, and 4. See the companion instrument papers for EMIRS (Edwards et al. 2021), EXI (Jones et al. 2021), and EMUS (Holsclaw et al. 2021) for details, also included in this special issue.

### Science Instruments

The three science instruments on the Hope probe are described in the subsections below. Table 1 provides summary data about each instrument. Each instrument is described more thoroughly in companion papers by Edwards et al. (2021), Jones et al. (2021), Holsclaw et al. (2021).

**Table 1:** EMM instrument summary

	EMIRS	EXI	EMUS
Mass	14.715 kg	16.92 kg	22.3 kg
Power	22.24 W	31.9 W	<13 W
Field-of-view	5.4 mrad	18.6° (UV)	10.75°
		25.8°×19.3° (VIS)	
Dimensions		*Sensor Head*	*Spectrograph*
	52.9×37.5×34.6 cm	32.77×36.07×39.88 cm	73×50×22 cm
		*Ebox*	*Ebox*
		8.05×26.54×26.67 cm	24×25×10 cm

#### EMIRS

**Fig. 4:**
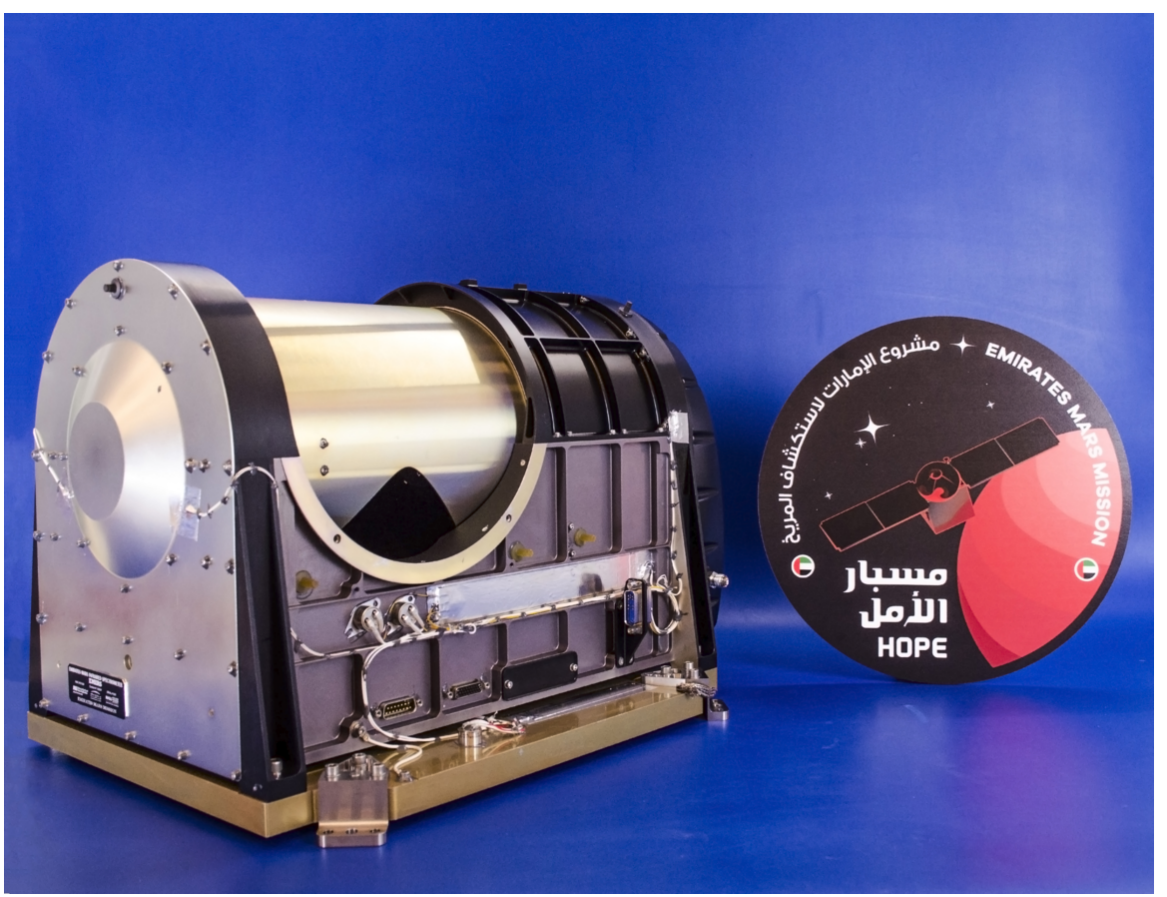
The EMIRS flight instrument

The Emirates Mars InfraRed Spectrometer (EMIRS, Fig. 4) is a Fourier transform infrared spectrometer that captures synoptic views of the martian disk over ∼1/2 hour of observing. EMIRS measures the infrared spectrum in ∼150 and 300 spectral wavelengths from 1666 to 100 cm^−1^ (6-100 μm) in 10 and 5 cm^−1^ spectral sampling. The instrument has mass of 14.715 kg and average power consumption of 22.24 W. The EMIRS instrument was built at Arizona State University (ASU). It leverages a long heritage of instruments led by ASU, including the Mars Observer and Mars Global Surveyor Thermal Emission Spectrometers (MO-TES and MGS-TES (Christensen 1992; Christensen et al. 2001)), the Mars Exploration Rover (MER) Miniature-Thermal Emission Spectrometers (Mini-TES), (Christensen et al. 2003) and most recently the OSIRIS-REx Thermal Emission Spectrometer (OTES, (Christensen et al. 2018)). EMIRS is the next generation in this lineage, incorporating and expanding on design updates from OTES, while returning the MO/MGS-TES pointing mirror capability to flight. Furthermore, the updated electronics of EMIRS, allow for a more complex commanding scheme than previous instruments but also permit on-board, lossless compression of the interferogram science data, self-safing due to sun impingement in the field of view, and enhanced servo performance to reject spacecraft induced vibrations and improve the disturbance rejection associated with the EMIRS pointing mirror impacts on the EMIRS servo. These capabilities enable the EMM concept of operations where the EMM spacecraft provides a single axis of movement to slew across the martian disk, while the EMIRS pointing mirror scans back and forth to build a 2-D image of the martian disk.

EMIRS has an Instantaneous Field of View (IFOV) of ∼5.4 mrad, enabling surface footprints of <300 km/pixel over the 0-70° emission angle range where retrievals of atmospheric properties can be made. The EMIRS pointing mirror is a 45° fold mirror that feeds the collected light into a 17.78 cm diameter f/3.3 Ritchey-Chretien Cassegrain telescope. This telescope feeds the light into the EMIRS interferometer, which is a Michelson design that leverages a moving mirror and fixed mirror that produces constructive and deconstructive interference that is measured by the EMIRS detectors. The moving mirror position and velocity are controlled by a laser metrology assembly and servo feedback loop with an infinite life flexure linear actuator. The $3\times 3$ array of detectors are uncooled pyroelectric Deuterated L-alanine doped Triglycine Sulfate (DLaTGS), though only the center detector meets the performance requirements across the full wavelength range and is all that is needed to meet science requirements. EMIRS uses a full aperture, two-point calibration to meet absolute radiometric performance of <1.5%. Specifically, EMIRS uses periodic observations of an internal calibration target and observations of space that bracket each observation column to provide a robust calibration methodology.

Over an EMM orbit around Mars, EMIRS will make up to 20 observations of about half of the Martian disk within half an hour of observation, including space and internal calibration observations. Using these infrared observations from 1666 to 100 cm^−1^ (6-100 μm), EMIRS will determine the of the column integrated abundance of atmospheric water vapor, the column integrated dust and water ice opacities (e.g. Smith 2002), and the atmospheric temperature profile as derived using the $\mathrm{CO_{2}}$ absorption feature at ∼15μm (e.g. Conrath et al. 2000). These parameters are retrieved from the infrared spectrum using a radiative transfer forward model following the approach used for MGS-TES (Conrath et al. 2000; Smith et al. 2002). Specifically, EMIRS will make global observations of the state of the lower martian atmosphere on sub-seasonal times scales over all local times. These observations, in concert with those of EXI and EMUS will enable the robust determination of the effects of the state of the lower atmosphere on atmospheric escape.

EMIRS will produce numerous data products useful for the scientific community. The EMIRS calibrated radiance for each pixel is stored in a Level 2 data product in Planetary Data System (PDS)-compliant Flexible Image Transport System (FITS) files with geometry that describes the spatial position of each EMIRS footprint included. In addition to these data products, quick look products of brightness temperatures at set wavelengths illustrate the geometry under which the observations will also be produced. From every pixel of <70° average emission angle we will retrieve the dust opacity, water ice opacity, column-integrated water vapor abundance, as well as atmospheric temperature profiles to 60 km, surface temperature and surface emissivity. The aforementioned retrieved parameters are stored and archived in level 3 FITS files of the same geometry as the level 2 products. In addition, re-gridded data products will also be generated. These products will grid individual observations into eight 3-hour local time bins over the previous ∼5° $\mathrm{L_{S}}$ over the full globe. These re-gridded products, along with the map projection/geometry are stored as PDS-compliant FITS files.

#### EXI

**Fig. 5:**
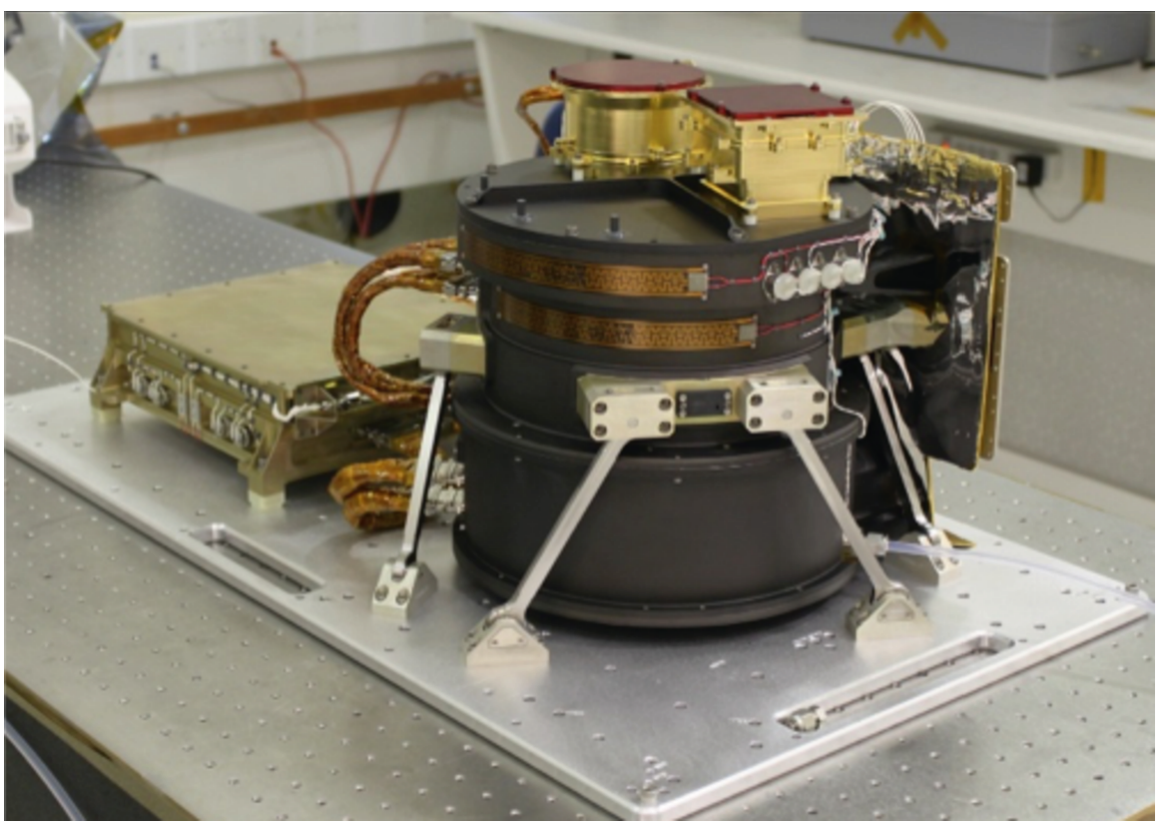
EXI flight instrument

The Emirates eXploration Imager (EXI, Fig. 5) is a 16.92 kg, 31.9 W dual-telescope imaging system that provides full disk views of Mars using six bandpasses (220, 260, 320, 437, 546, 635 nm). The use of two optical paths is necessary to maintain high quality images across the spectral coverage of EXI, where the ultraviolet (UV) channel accommodates the first three bands and the visible (VIS) channel includes the last three. Both optical paths share a common filter wheel, where the thickness of the filter is used to optimize the focus. In addition, each set of optics includes a baffle system to eliminate effects associated with reflected sunlight, including glint from the nearby surface of the spacecraft. Each telescope uses a CMOSIS CMV 12000 detector with a format of 4096×3072 pixels, i.e. 12.6 megapixels. The detectors are backside-illuminated devices, but without the typical micro-lenses and color filter arrays (i.e., Bayer filter).

The EXI IFOV is determined by a combination of the detector pixel size (5.5 μm) and the lens system. The UV telescope has a focal length 47.5 mm with f/3.6, while the VIS system has a focal length of 50.6 mm and a slower speed of f/4.25. For the VIS system, this slower f/stop is associated with the much higher irradiance in Martian reflected light for the visible wavelengths compared to those of the UV. The parameters combine to give a circular field of view (FOV) of 18.6° for the UV and a rectangular 25.8°×19.3° FOV for the VIS. This ultimately provides better than 24.8” and 24.0” per pixel for the UV and VIS telescopes, respectively. These IFOV values give a single pixel footprint of 2-4 km from periapsis to apoapsis.

The design of EXI was driven by the desire to characterize the distribution of atmospheric constituents in the lower atmosphere such as water ice particles and ozone. Such observations are done synoptically and with mesoscale resolution (i.e. 10-100 km). By combining the EMM orbit with planetary rotation, EXI provides both global (spatial) and diurnal sampling of Mars on sub-seasonal timescales. In other words, EXI images capture both diurnal and seasonal timescales and associated variations for a large fraction of the planet. The derivation of these atmospheric quantities will employ primarily the 260, 320, and 635 nm bands using a look-up table (LUT) based radiative transfer analyses similar to that developed for the Mars Color Imager (MARCI) onboard the Mars Reconnaissance Orbiter (MRO) (Malin et al. 2008; Todd Clancy et al. 2016; Wolff et al. 2019).

EXI will produce data products intended for use by the global scientific community. Each image take will be provided with units of calibrated radiance and with the associate geometric information (i.e., longitude, latitude, photometric angles) as part of the Level 2 product. In addition, the water ice cloud optical depths and ozone column abundances will also be generated in image format for all pixels with incidence and emergence angles less than 70° (i.e., within the validity of the plane parallel approximation). These data will be delivered in the PDS-compliant FITS data format with multiple Header/Data Units (HDUs) to provide both the primary image quantities and associated metadata.

#### EMUS

**Fig. 6:**
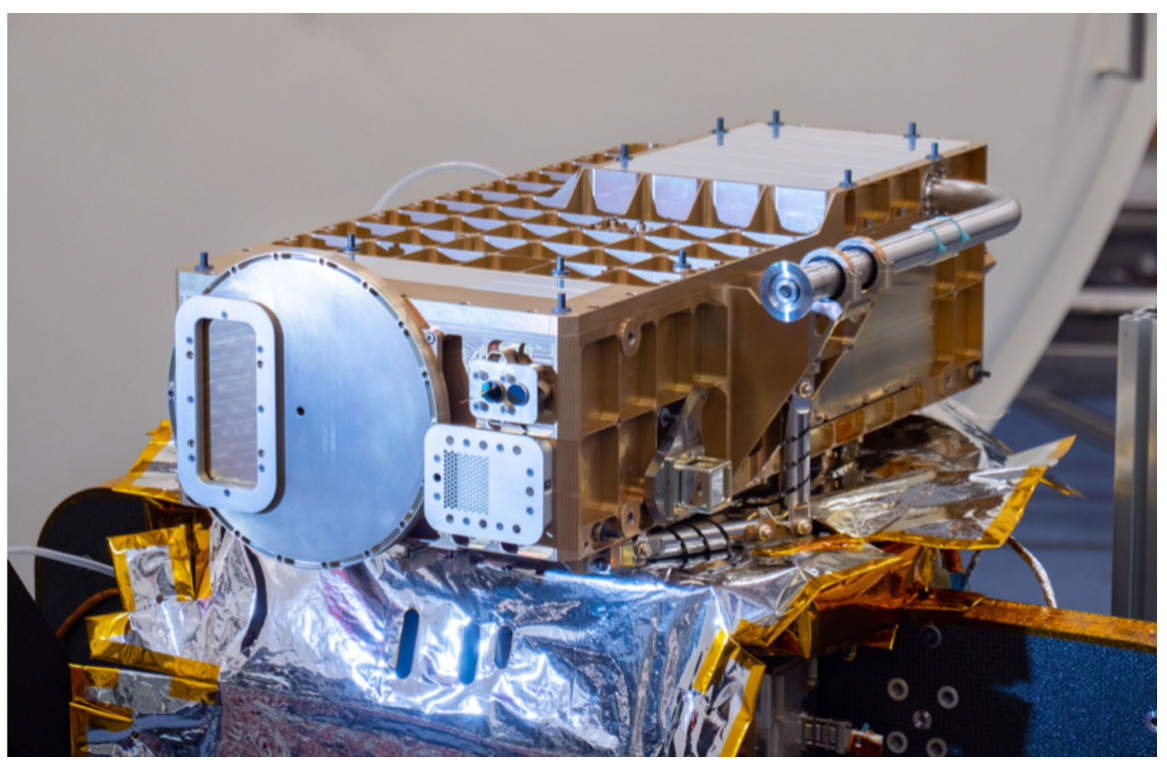
EMUS flight instrument

The Emirates Mars Ultraviolet Spectrometer (EMUS) consists of two components: 1) the “spectrograph”, which includes the optical channel, detector and its electronics, and high voltage power supply (HVPS) and 2) the electronics box (Ebox), which includes three boards connected by a common backplane: power, channel, and processor board with the FPGA. The Ebox is mounted on the main instrument panel with EXI, EMIRS, and the star trackers. The spectrograph is mounted to a subpanel with titanium struts arranged in three bipods. The total mass of the EMUS instrument is 22.3 kg, the orbit average power is less than 13 W, and includes an estimated maximum of 4.6 W of proportional heater control. A picture of the EMUS spectrograph is shown in Fig. 6.

The optical design of EMUS is based on a two-element imaging spectrograph, similar to previous instruments such as Cassini UVIS (Esposito et al. 2004). A reflective, spherically-figured telescope mirror with a focal length of 150 mm images the scene onto a long-slit aperture that defines the EMUS field of view (FOV). A rotational slit-change mechanism allows the selection of one of four slits of equal height, subtending 10.75°, and varying width that provide spectral resolutions of 0.35 nm (very high or VHI), 1.3 nm (high or HI), 1.8 nm (low or LO), and 5 nm (very low or VLO). Only the HI and LO slits are currently planned for routine science observations. The spectrograph design is based on a Rowland circle configuration and consists of a reflective, diffraction grating that disperses and images the entrance slit onto the detector. The grating is toroidally figured to reduce astigmatism, and the laminar facets are ion-etched directly into the fused silica substrate with a ruling density of 936 gr/mm ruling.

To enable high throughput across the required wavelength region of 100-170 nm, we make use of area-division optical coatings in a common optical path. Silicon carbide (SiC) provides a normal-incidence reflectance of 40% at 100 nm (Soufli et al. 2009), while reflective aluminum with a protective overcoat of magnesium fluoride (together, $\mathrm{Al}+\mathrm{MgF}_{2}$) provides 90% reflectance at 170 nm. Half the area of each optic is coated with SiC while the other half is coated with $\mathrm{Al}+\mathrm{MgF}_{2}$. The dividing line between the two coatings is centered on the optics and parallel to the entrance slit; thus, the projected beam from the aperture stop, located in front of the telescope mirror at a position that forms a conjugate image at the grating, illuminates nearly equal areas of each coating at all field angles due to the narrow angular subtended by the science slits.

The EMUS detector is a photon-counting, open-face microchannel plate (MCP) device with a cross-delay line (XDL) photon-locating anode provided by the University of California, Berkeley Space Science Laboratory. The 3-element MCP “z-stack” is coated with an opaque cesium iodide (CsI) photocathode. The circular 38 mm diameter active area of the detector is defined by a thin metal aperture placed between the bottom two MCPs in the stack. Electrons are accelerated down the MCP pores by a high voltage potential maintained across the MCPs. Interaction of these electrons with the walls of the MCP pores results in stochastic amplification process, with an ultimate gain determined by the voltage applied across the MCPs and set to a modal value of ∼1 pC (6.2e6 e-) to balance detector lifetime and electronics resolution capabilities. Photoevents are processed individually with a position recorded to 12 bit precision and an accuracy of 0.08 mm. Native pixels greatly oversample the smallest required spectral × spatial resolution element of 1.3 nm × 0.36°, and so the detector is typically binned 8×16 for a resulting sampling of 0.5 nm × 0.16°.

A reclosable aperture door at the front of the EMUS telescope opens for science observations and closes between activities for protection against potential damage from accidental pointing toward the Sun.

### Science Orbit and Observation Coverage

The EMM Science orbit has been developed to provide a consistent vantage point for the instruments to collect near-global, diurnal maps of the Martian system every 9-10 days. The science orbit is illustrated in Fig. 1, and has the following characteristics:

- The orbit has a mean periapse altitude of approximately 20,000 km, a mean apoapse altitude of approximately 43,000 km, and an orbital period of approximately 55 hours. This amounts to an orbital period of ∼2.25 Martian sols, meaning that every revolution about Mars places periapse  of a rotation about Mars and every 4 revolutions about Mars completes a map.
- The orbit has an inclination of 25° relative to the Martian pole. This permits observations to observe the poles while providing substantial diurnal mapping of the majority of the Martian system.
- It has an argument of periapse that sweeps from 177 – 183° during the mission. This balances Northern and Southern mapping.
- The local time of periapsis precesses at the same rate that Mars moves about the Sun, so that high spatial resolution observations are made at all local times over the course of a Martian year.

**Fig. 7:**
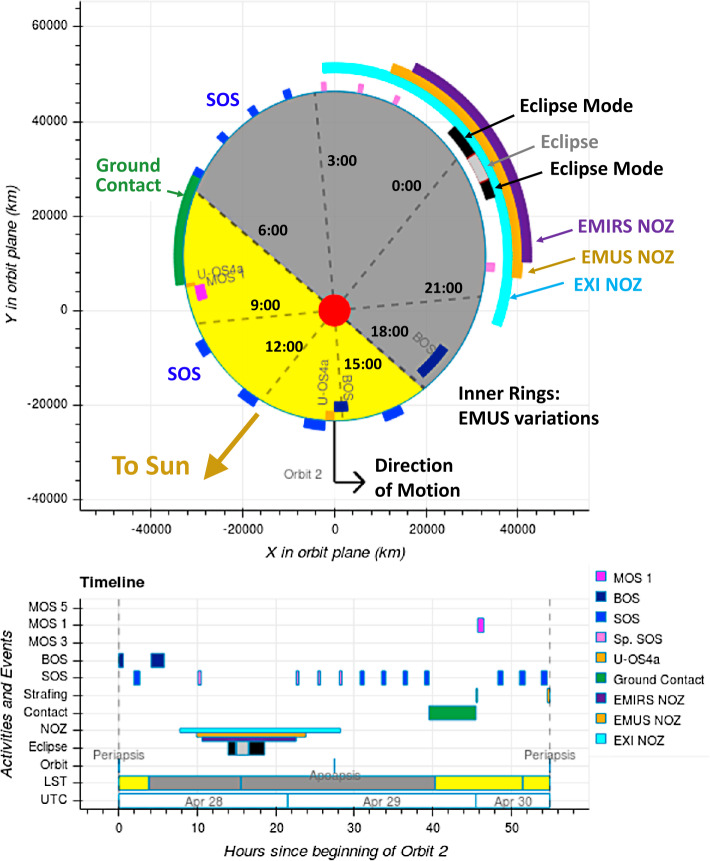
Diagram of an “orbit in the life” of EMM, with sample color-coded observation sequences, NOZ locations, and ground contact indicated as viewed from above Mars (top) and on an orbit timeline (bottom). The orbit start and stop location is at midnight LST, and yellow and gray shading indicate the sunlight and shadowed parts of Mars

The concept of operations (ConOps) for the spacecraft has been constructed to enable the collection of scientific observations needed to meet the science objectives of the mission from this orbit. To illustrate the ConOps, an “orbit in the life” of the Hope probe is shown in Fig. 7.

Each of the three instruments performs a variety of remote sensing observations of the planetary system; most are centered about the nadir and extend some angular distance from nadir. None of the instruments can safely point at the Sun, therefore the ConOps protects them via “No Nadir Observation Zones” (NOZ): each NOZ is sized according to the requirements of each instrument and its observation: the EMIRS NOZ is approximately 55.1°. While it’s possible for an infrared mapping observation to be off-centered from the nadir, the ConOps expects each observation to be centered about nadir. Accounting for this as well as potential pointing errors, the spacecraft’s position knowledge error and uncertainty, and many safety allocations, EMIRS observations must be placed at least 55.1° away from the Sun-Mars line. Similarly, the EMUS NOZ is sized to be 65.9°and the EXI NOZ is sized to be 94.8°. EMUS has many different observation strategies, including observations that extend not far from nadir and observations that extend well away from nadir; these are accounted for individually. The solar panels and High Gain Antenna on Hope are not gimbaled, and the spacecraft therefore turns to point the HGA toward Earth during ground contacts. Science observations are not made during this time. Between observations, the spacecraft points the solar panels toward the Sun.

The ConOps is built to support a routine cadence of EMIRS observations such that the spacecraft performs an EMIRS scan approximately once every 2.5 hours with a battery recharge between observations. When the EMIRS scan is outside of the EXI NOZ, then an EXI image is collected at the beginning and end of the scan, where the first is higher resolution than the second. This EXI-1 + EMIRS + EXI-2 sequence is known as a Small Observation Sequence (SOS). The SOS is the fundamental building block of the EMM ConOps.

To maximize efficiency in the ConOps, EMUS observations are added to SOS activities, either by placing one or more EMUS observations at the beginning, end, or throughout the SOS. Observations are placed to align them with ideal locations in the Mars-Solar-Orbital (MSO) frame, where the pole aligns with Mars’s orbital pole and the x-axis points from Mars to the Sun. Thus, the sub-solar point lands on Mars on the MSO equator and prime meridian, though that may be up to 25°away from Mars’s actual equator, depending on the season.

EMUS has five different types of observation sequence as follows and as described in the companion paper by Holsclaw et al.:

- EMUS Type I observations (U-OS1) are composed of two scans across the Martian limb, each extending from 0 to 1.06 $R_{M}$ from nadir and spaced (ideally) $+/-$ 60° apart in MSO longitude. These observations will be used to determine the relative abundance of oxygen and carbon monoxide in the thermosphere.
- EMUS Type II observations (U-OS2) are similar to U-OS1 observations, but scan to larger altitude (1.6 $R_{M}$) in order to determine the distribution and variability of hydrogen and oxygen in the Martian exosphere. These observations are placed around an entire spacecraft orbit.
- EMUS Type III observations (U-OS3) consist of four large observation swaths where the spacecraft slews out to ±50° in an asterisk pattern. The scientific goal of these observations is the same as for U-OS2 - measurement of the hydrogen and oxygen exosphere.
- EMUS Type IV observations (U-OS4) look across the spacecraft orbit (not toward Mars) through the mid and outer corona. They have long exposure times, and are paired with observations placed ‘across’ the orbit with the boresight oriented in the same direction, to remove background from the original observation.
- EMUS Type V observations (U-OS5) are calibration activities.

The observation sequences have been labeled as either small (SOS), medium (MOS), or big (BOS), indicating their complexity. The SOS was described above and has two main variations: one that includes EXI images and one that does not. The Big Observation Sequence is one that includes U-OS2 observations; these observations are time consuming and may drain the battery more than other observations. All other EMUS activities are part of Medium Observation Sequences. A notable exception are observations made from within the EXI NOZ, when only EMIRS observations are made; these are referred to in the ConOps as Special SOS (Sp. SOS in Fig. 7).

Hope’s ConOps retains the flexibility to define and implement new observations outside of the SOS/MOS/BOS framework. Examples could include observations made for public relations purposes, or to enable new science identified by the science team.

The ConOps balances all of the resources available on the spacecraft, which mandates that two BOS observations are separated by a SOS, a SOS separates a ground contact from a BOS, and other similar requirements. Thus, observations are not always placed precisely where they are ideal, but are placed such that the science coverage is maximized. Further, the observations do not simply satisfy the science requirements but work to maximize them within the resources and constraints in the system.

### Science Analysis

**Fig. 8:**
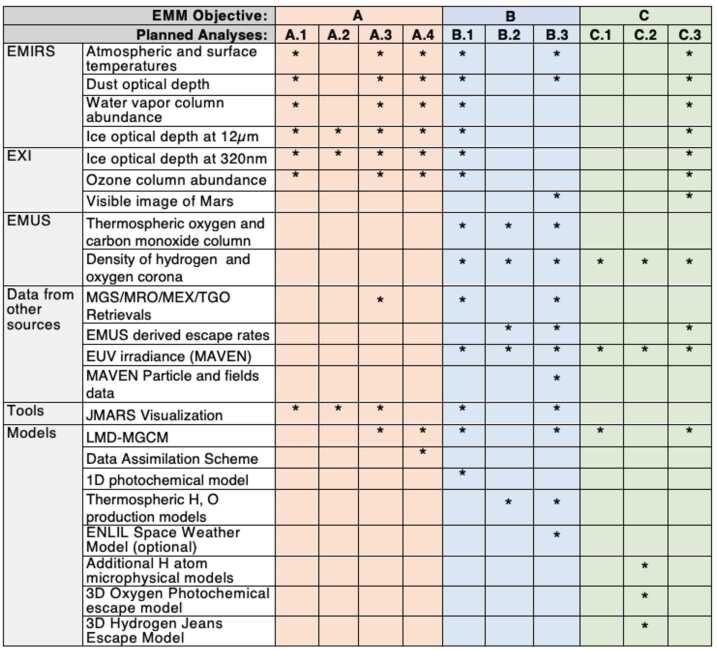
Mapping of the analyses for the EMM science objectives to the required EMM data, and other data, tools, and physical models

EMM will characterize Martian atmosphere dynamics and processes by achieving three scientific objectives (Sect. 2.2) through analysis of data from three scientific instruments (Sect. 3.2) observing the lower atmosphere, the thermosphere, and the exosphere of Mars geographically over both diurnal and seasonal timescales. The subsections below summarize the EMM science team’s strategy for each objective in terms of required analyses, and Fig. 8 maps the data, tools, and physical models required for each analysis. A detailed description of EMM science analysis plans designed to satisfy (close) the mission’s science objectives can be found in the companion paper by Almatroushi et al. (2021).

#### Objective A (Lower Atmosphere) Analysis Strategy

Using data from the EXI and EMIRS instruments, EMM will characterize the state of the lower atmosphere of Mars by determining the structure and variability of atmospheric and surface temperatures and the geographic and diurnal distribution of dust, water vapor, water ice, and ozone. This will aid in understanding the energy balance in the current Martian climate and how the lower atmosphere responds to solar forcing diurnally and seasonally. The planned analyses to fulfill objective A are:

1. Merge observations into a combined multi-dimensional snapshot of the global atmosphere with respect to altitude, latitude, longitude, local time, and season
2. Compare products of similar data quantities between EXI and EMIRS to aid in cross-calibration
3. Conduct spatial and temporal comparisons to global climate models such as the LMD-MGCM (e.g. Forget et al. 1999) and other observations or spacecraft datasets
4. Ultimately produce a reference climatology for Mars using meteorological data assimilation techniques (e.g. Navarro et al. 2017)

#### Objective B (Lower-Upper Atmosphere Correlation) Analysis Strategy

EMM will correlate the rates of thermal and photochemical atmospheric escape with conditions in the collisional Martian atmosphere utilizing all three EMM instruments and by performing the following analyses:

1. Correlate conditions in the lower atmosphere with those in the upper atmosphere to better understand lower-upper atmosphere connections
2. Compare escape rate variations (and other exospheric properties) with thermospheric conditions (including $\mathrm{CO/CO_{2}}$ and $\mathrm{O/CO_{2}}$ column density ratios) to probe how the transition from collisional to collisionless regimes moderates neutral escape
3. Analysis of the response of the atmosphere and escape rates to episodic events, specifically dust storms, solar flares, solar energetic particle and coronal mass ejection events, polar ice cap variability, and dust deposition and removal events

#### Objective C (Exosphere) Analysis Strategy

EMM will characterize the spatial structure of hydrogen and oxygen density and escape in the Martian exosphere and their variability with respect to season and solar activity. This will be achieved using densities and temperatures derived from UV brightnesses observed by the EMUS instrument and will help to elucidate the processes that govern exospheric dynamics and escape. The planned analyses to fulfill objective C are:

1. Compare hydrogen and oxygen derived density structures (i.e. with respect to local time or latitude) to predictions of these structures from global atmospheric models
2. Derive escape rates from exospheric density profiles using a range of techniques, from simple Chamberlin exosphere calculations to detailed micro-physical escape models
3. Somewhat analogously to C.1, compare EMUS-derived escape rates at a given time to predictions of escape rates calculated by models incorporating the simultaneous lower-atmospheric conditions measured by EXI and EMIRS

## Mission Plan

### Operational Mission

The EMM operational mission is separated into operational phases, each of which has specific objectives and exit criteria. Prior to beginning science observations at Mars, the project must:

1. Test and characterize the in-flight performance of the spacecraft and instruments
2. Execute maneuvers to target the Mars arrival point
3. Perform the Mars orbit insertion maneuvers
4. Further calibrate instrument in Mars orbit
5. Perform maneuvers to shape the orbit to the correct science orbit

**Fig. 9:**
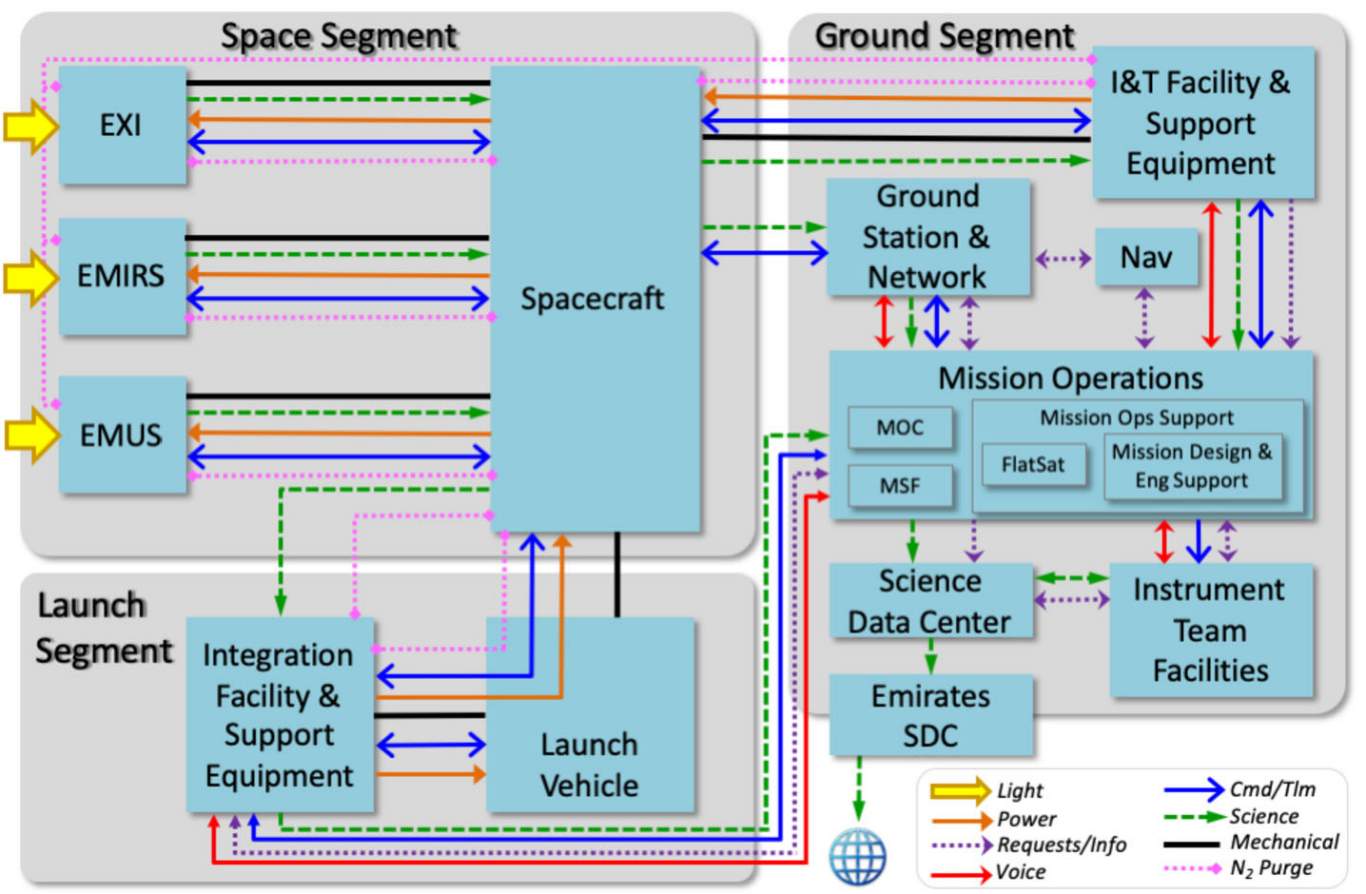
Block diagram showing the relationship between the launch, space, and ground segments of EMM

Figure 9 shows how the space, ground, and launch segments of the mission connect to each other, while Fig. 16 shows the operational phases, milestone reviews, and significant maneuver events.

#### Mission Design

The Emirates Mars Mission was designed to traverse a Type I interplanetary transfer to Mars, launching between July 14, 2020 UTC and August 12, 2020 UTC and arriving at Mars on February 9, 2021 UTC. The mission was designed to minimize risk and complexity while delivering the vehicle to the target science orbit as described in Sect. 3.3. This involves launching on a direct transfer to Mars, biased for planetary protection. A total of six Trajectory Correction Maneuvers (TCMs) were planned to systematically remove the planetary protection bias and target the arrival aimpoint. Upon arrival at Mars, the spacecraft executes the Mars Orbit Insertion (MOI) maneuver, which transitions it from its hyperbolic arrival to an elliptical capture orbit. Finally, the spacecraft executes three Transition to Science Maneuvers (TSMs) to efficiently shift from the initial capture orbit to the target science orbit. Planetary protection is fully satisfied from a navigation perspective by maintaining sufficiently low probabilities that the spacecraft and launch vehicle are ever on impact trajectories with Mars.

#### Mission Phases

**Fig. 10:**
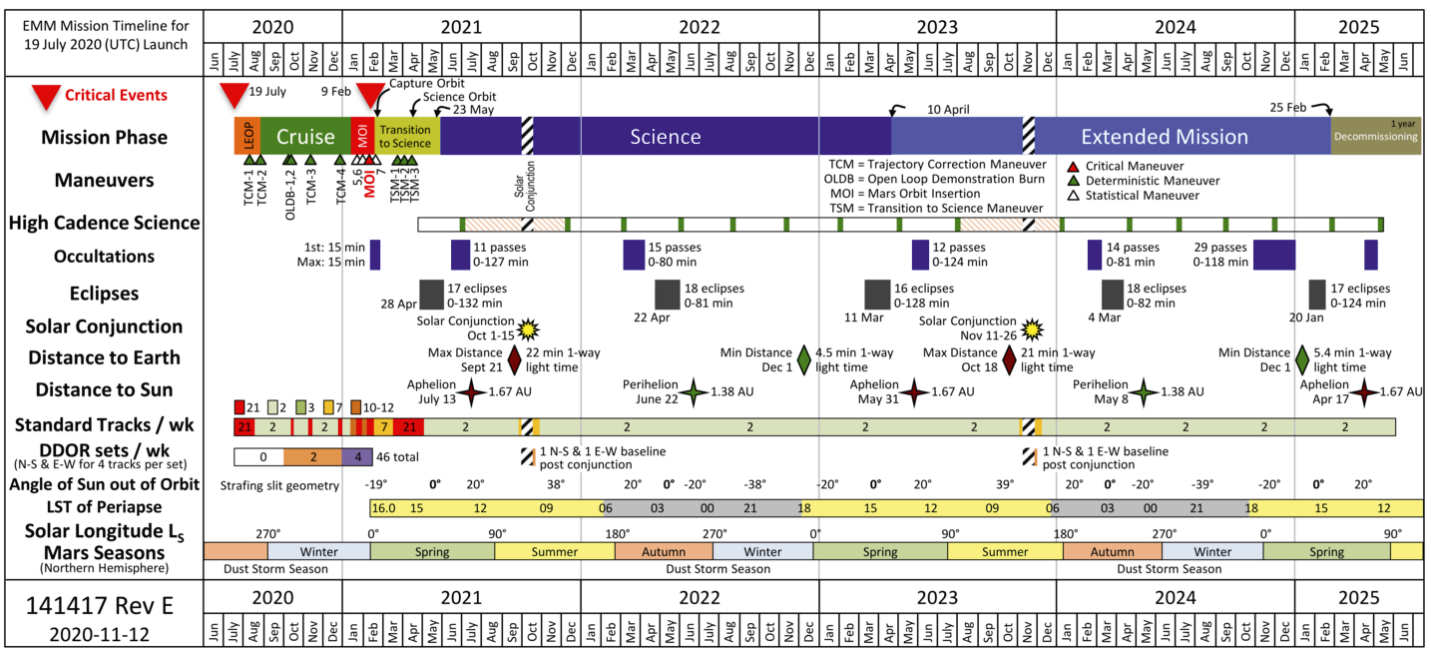
Mission timeline showing mission phases, various mission events such as planned maneuvers and eclipse and solar conjunction periods, as well as information such as available data download volume and Martian season

The operational mission is separated into phases with distinct objectives, as shown in Table 2, Fig. 10, and discussed below.

**Table 2:** EMM operational mission phases

Operational phase	Start date	Objectives
Launch	19 July, 2020 (UTC)	Captured the final spacecraft configuration and power down for flight. Executed countdown and launch. Performed separation from the LV and initial power on, which included the configuration necessary to communicate health and safety to the Ops Team.
Early Operations	19 July, 2020	Performed initial commissioning of the spacecraft subsystems and aliveness testing of the science instruments, and executed 2 TCMs.
Cruise	1 September, 2020	Began at EOP termination and lasted until 30 days before MOI.
Mars Orbit Insertion	10 January, 2021	Began after Cruise completion and lasted until Navigation confirmed the Observatory was in a stable orbit.
Transition	10 February, 2021	Began at MOI phase termination and lasted until the Observatory was in an acceptable science orbit and commissioning was complete (approximately 75 days).
Science	23 May, 2021	Began at Capture/Transition phase termination and lasts for 1 Martian year (687 Earth days)
Extended Mission	10 April, 2023	Begins after a successful Science Phase with duration dependent upon a number of factors
Decommissioning	TBD	Begins at the end of the Extended Mission on a date established at the Decommissioning Review (DR) and lasts until all observatory decommissioning and project closeout activities are complete

##### Launch Phase

EMM utilized a 30-day Launch Period, 14 July, 2020 to 12 August, 2020 (UTC), launching from Tanegashima Space Center, Japan, on a Mitsubishi H-IIA-202 launch vehicle. After several scrub events due to poor weather at the launch site, EMM launched on 19 July, 2020 (UTC). EMM utilized a Type 1 trajectory to Mars, scheduled to arrive on 9 February, 2021, regardless of the launch date. No launch commit criteria were violated during the countdown process, and the launch trajectory met the mission needs. Once the Hope Probe separated from the upper stage, an automated sequence began to awaken the probe. The central computer booted up and immediately turned on heaters to prevent the fuel from freezing. It then deployed the solar array panels, and leveraged the sun sensors to maneuver to the Sun so that the solar arrays could begin charging the battery. At that point Hope began to send transmissions to the Earth, where the NASA Deep Space Network (DSN) ground station in Madrid captured lock. Once the Hope Probe was in contact with the ground station, the EMM Operations Team checked the health of the spacecraft.

##### Early Operations Phase

The Early Operations Phase (EOP) was a 45-day period that checked out the various spacecraft subsystems for functionality and performance characteristics. This included commissioning of the telecommunications, attitude determination and control, propulsion, electrical power and distribution, thermal, flight software, command and data handling subsystems. The first Trajectory Correction Maneuver of the mission (TCM-1) was accomplished on 11 August 2020 UTC, and removed 10 m/s of launch bias from the trajectory without causing the delivered aimpoint and 3-sigma dispersion to cross the Mars impact zone. The subsequent TCM-2 completed the majority of the launch bias removal. EMIRS accomplished initial power on and aliveness testing, EMUS performed initial power on and checkout, and EXI completed the electronics box checkout. The phase concluded with the successful completion of the Post-Launch Assessment Review (PLAR), where the functionality and performance of the observatory was reviewed for readiness to enter Cruise Phase.

##### Cruise Phase

The Cruise Phase incorporated the five months between EOP and the Mars Orbit Insertion (MOI) Phase. The objective was to further characterize the science instruments, to execute two more TCMs, and to prepare for Mars orbit insertion. Instrument activities included stellar calibrations, further characterization, and observation demonstrations.

##### Mars Orbit Insertion Phase

The objective of this phase was to obtain an orbit around Mars that met planetary protection requirements. MOI Phase began 30 days prior to MOI, and included a moratorium on extraneous observatory activities to allow the team to focus on MOI with known and demonstrated system configurations. The MOI maneuver was performed on 9 February 2020 (UTC) with the successful entry of Hope into Mars orbit. Two additional TCMs were possible in this phase. The MOI burn lasted for approximately 27 minutes and reduced the spacecraft velocity by ∼958 m/s, expending nearly half of the onboard fuel. The maneuver was completely autonomous and, due to one-way light time of ∼10.6-min, ground intervention due to an anomaly was not feasible. By design, the entire MOI occurred when Hope was in contact with Earth, at the expense of a small amount of fuel.

##### Transition Phase

Over ∼75 days following MOI the first images of Mars from EMM orbit were taken, the operations team began practicing the science observations, and maneuvers were performed to place Hope into its science orbit. The orbit was re-shaped from ∼1,000 km × ∼49,000 km to ∼20,000 km × ∼43,000 km, meeting the requirements in the STM described in Sect. 3.1.

##### Science Phase

Hope completes one orbit of the planet every 55 hours, making science observations as described in Sect. 3.3. Contacts occur twice per week for six hours each. The mission plans to receive over 1 terabyte of novel data on Mars’s atmosphere and its dynamics over the course of one Martian year.

## Mission Team

The Emirates Mars Mission presented a number of unique challenges to forming, organizing and managing a team. Chief among these challenges was the geographic separation of a team that needed to be, as an inherent aspect of the program goals, tightly integrated at every level. This geographic separation was evident in time-of-day phasing issues as well as institutional and cultural differences. While it was necessary for each organization to adhere to the local customs and constraints of that organization, mission success depended on these teams forming a whole and integrated team. The technical needs of the program also presented a challenge to forming the team as neither organization had the experience needed in all areas of deep space exploration to accomplish the programmatic goals. It was therefore necessary to hire a significant number of experienced personnel and integrate them into the existing organizational structures in a very short time so as not to impact the accelerated development timeline. Finally, programmatic resources necessitated the formation of a small and efficient team structure, which had to effectively manage the 6 year development period of the program over which time the character, needs and priorities of the mission evolved dramatically. It was imperative that the organizational structure of the team was not only robust to these programmatic pressures, but also capable of supporting and enabling the capabilities of each individual contributor to their fullest extent. EMM’s mission team and organizational structure was formed to respond to these challenges, and has as its principal aspect a centralized management approach with decentralized execution.

### Mission Level

**Fig. 11:**
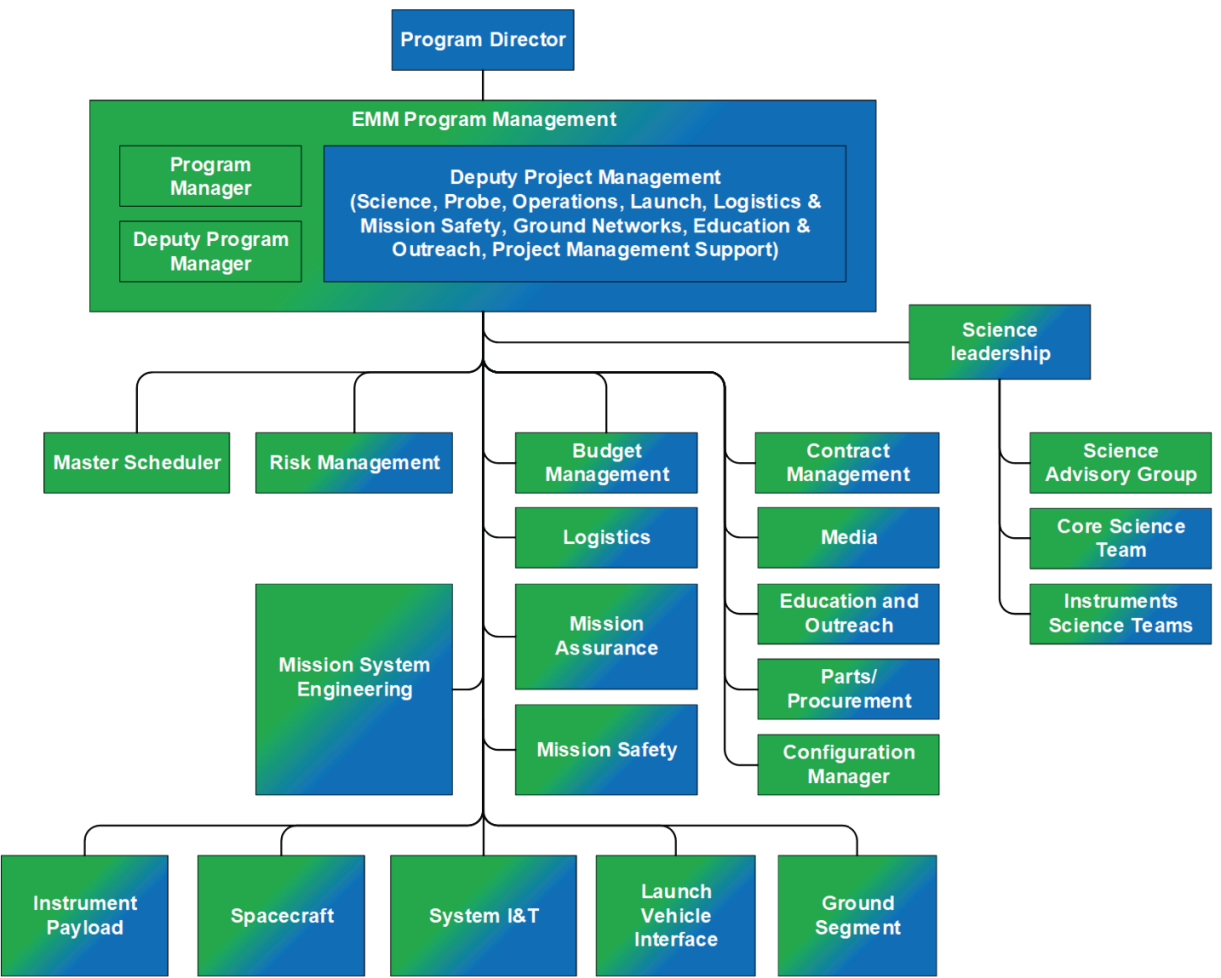
Mission level EMM organizational structure. MBRSC contributions are indicated in blue and Knowledge Transfer Partner contributions in green

In Fig. 11, the mission level portion of the EMM organizational structure is shown. The figure is color coded to more clearly show the tight integration of the international team, with MBRSC team members shown in blue, and Knowledge Transfer Partner team members in green. Where a role or function was filled by a combination of team members, the box is both green and blue.

Several key features of EMM’s organizational structure at the mission level can be seen in this figure. First, the highly integrated nature of the program is evident, with a combination of UAE and US personnel at all levels from manager roles, through team leads and to the individual contributor level. This deliberate inter-weaving of the teams throughout the organizational structure provided significant advantages as inter-institution communication was greatly facilitated at all levels of the program. Similarly, reporting from each element of program was both inclusive of all teams and performed efficiently as a result of this integration at all levels.

Another key feature is the clear lines of authority. The mission has a single program director, and the program management office (PMO) responds to this direction to provide clear direction and efficient decision-making to the rest of the team. The PMO has direct lines of authority to all elements of the mission. The crucial mission level roles such as mission system engineering and mission assurance are tightly coordinated to the team structure, directly reporting to program management, but deliberately not placed between the PMO and the development team along the line of direction and reporting.

Roughly 80% of the EMM team is represented by the lower five boxes in this mission organizational chart. Each major system of the development effort is included in this row: Instruments, spacecraft, system integration and testing, interfaces to the launch vehicle, and the ground segment which includes all ground elements of the operations efforts, interface to the DSN and the science data center. Each of these systems has one US and one UAE lead. Each lead pairs remain in close coordination, providing management and guidance to and reporting from their respective systems. This is a critical aspect of the decentralized execution of the program, coordinated by a centralized management team, as mentioned in the previous section.

### Science Team

**Fig. 12:**
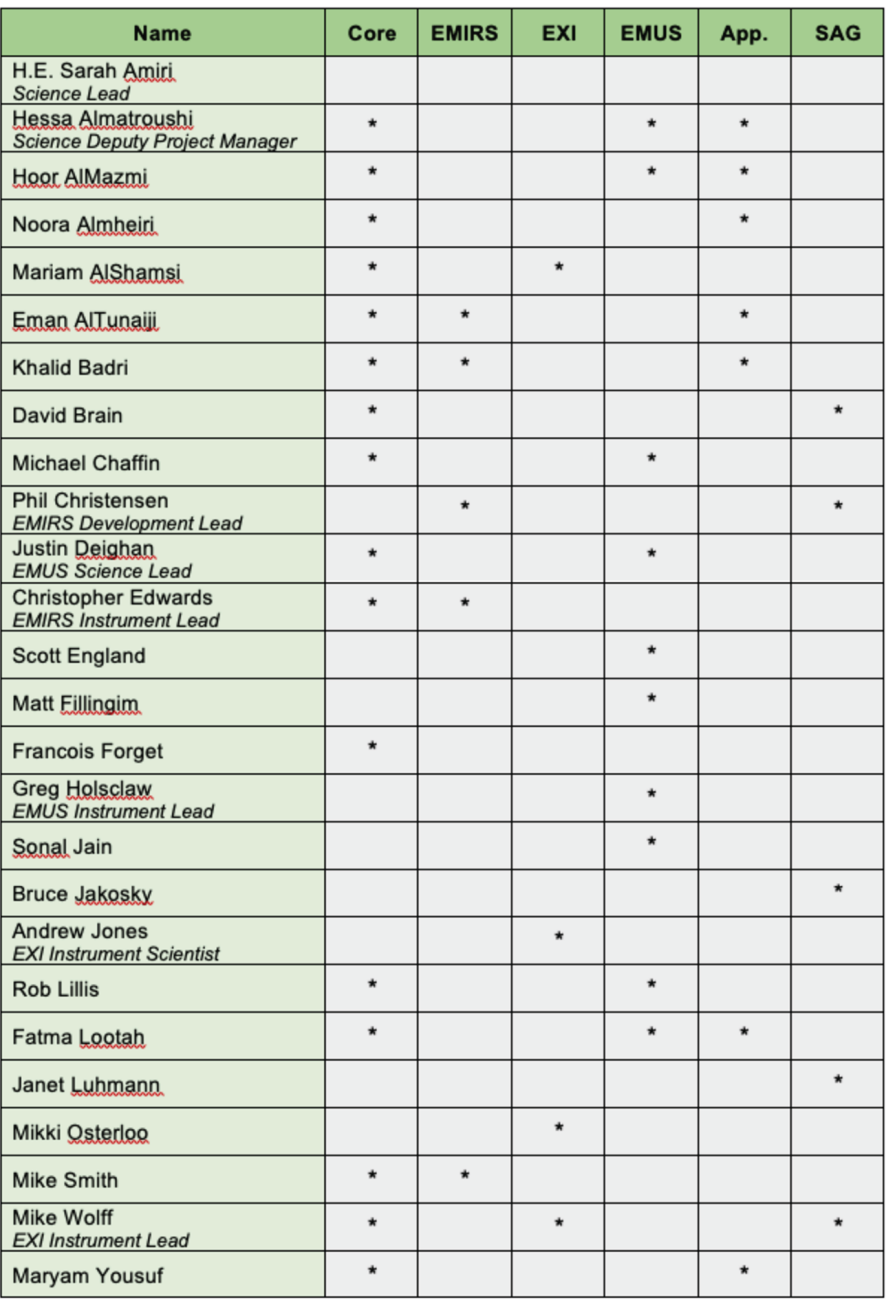
EMM Science Team members and their roles, including Core Science Team member, Instrument Science Teams, Apprentices, and the Science Advisory Group

The EMM science team has members from the UAE, U.S., and France, and its structure at the time of launch is shown in Fig. 12. A Science Leadership group, under the direction of the Science Lead, directs the science of the mission and represent the science team to program management. Each of the EMM instruments has an Instrument Lead and several team members. Four interdisciplinary scientists will work with data from multiple EMM instruments and/or relevant models. A Science Advisory Group (SAG) consisting of five EMM scientists experienced in mission or science management provides advice to the Science Lead upon request.

A unique aspect of the EMM Science Team is the role of Apprentice. As part of the programmatic objectives of the mission, several engineers from MBRSC have undergone a rigorous science apprenticeship program over the 4 years prior to launch. The engineers have worked closely with U.S. science team members on science analysis and data pipeline projects to prepare to work with EMM data when it becomes available. Apprentices have conducted scientific research at the level of graduate students, and presented the results of their work at international conferences.

In addition to the team members shown in Fig. 12, the EMM science team will also include students and postdocs from the participating science institutions, as well as participating scientists from universities in the UAE.

## Spacecraft

### Design

#### Overview

**Fig. 13:**
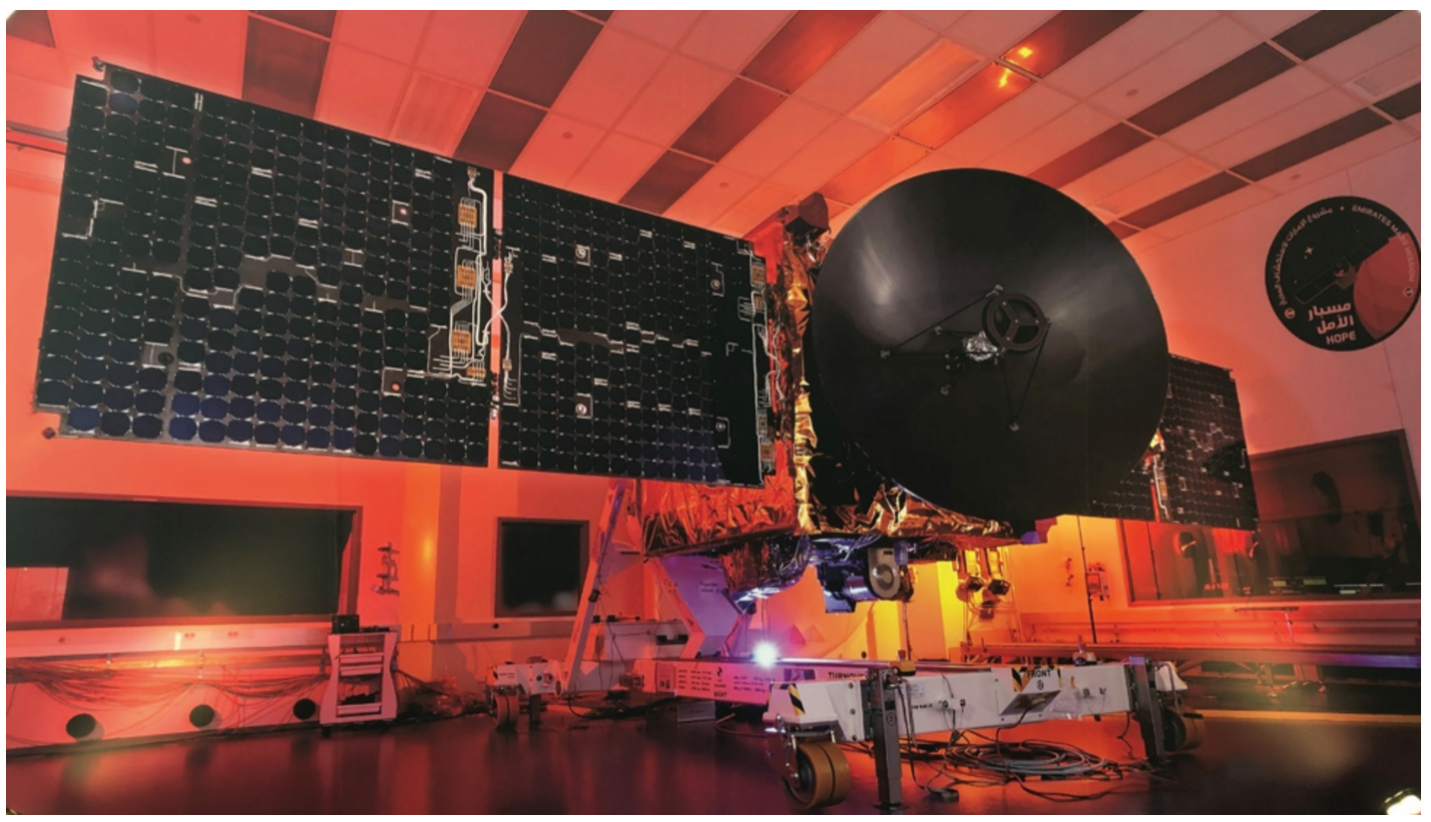
EMM Hope Probe undergoing solar array testing

The Hope Probe (Fig. 13) is a traditional three-axis stabilized, solar-powered spacecraft with a pressure-regulated hydrazine monopropellant propulsion system. It utilizes high gain and low gain antennas transmitting/receiving at X-band from a single radio and amplifier. Attitude knowledge is provided by two star trackers, eight coarse sun sensors, and two inertial measurement units. Attitude control is via four reaction wheels or the propulsion system. Thermal control is a mixture of passive and active, with active control achieved via thermostatic or software controlled heater circuits. The structure consists of a carbon fiber reinforced plastic thrust tube with an integrated launch vehicle adapter, and aluminum honeycomb panels with carbon facesheets. Tubular carbon struts are used to mount the bus and instrument panels to the central cylinder and a traditional clamp band system is used to attach the structure to the launch vehicle. The avionics are selectively redundant across the flight computer and the power control unit. The primary control software runs in a real time operating system with adaptable command sequences implementing mission-specific behaviors. A solid state recorder stores science and engineering data. An autonomous fault protection system provides robust anomaly handling. Observatory dry mass is approximately 543 kg and wet mass is approximately 1343 kg, and in the stowed configuration the Observatory is approximately 2 meters wide by 2 meters deep by 3 meters tall.

#### Architecture

The spacecraft uses a combination of custom-built and off-the-shelf parts. Specifically the flight computer, solid state recorder, structure, solar arrays, high gain antenna, harness, and flight software are custom components, while the balance of the components are catalog or modified versions of previous designs. This approach allowed the program to utilize its strengths and build capability while meeting the aggressive cost and schedule requirements of the mission.

The use of a selectively redundant architecture was another decision designed to balance risk and resources and grew out of a highly focused set of architecture studies that informed each component choice across the design. After studying more than 500 different failure modes, assessing historical failure data for candidate components, building complete Failure Modes (bottoms up) and Fault Tree (top down) analyses, and performing a vulnerability analysis, a spacecraft architecture with selective redundancy and six single point failures was developed. The simplicity of the design, the ability to perform extensive testing on every configuration of the vehicle, and the selection of highly screened parts for especially vulnerable components mitigates the risk. The additional use of two FlatSat test benches provides significant flexibility in testing and the ability to dry run both test and operational procedures before loading them to the flight vehicle. The FlatSats also allow extensive anomaly resolution and fault injection capability that supports the organization’s robust testing philosophy.

**Fig. 14:**
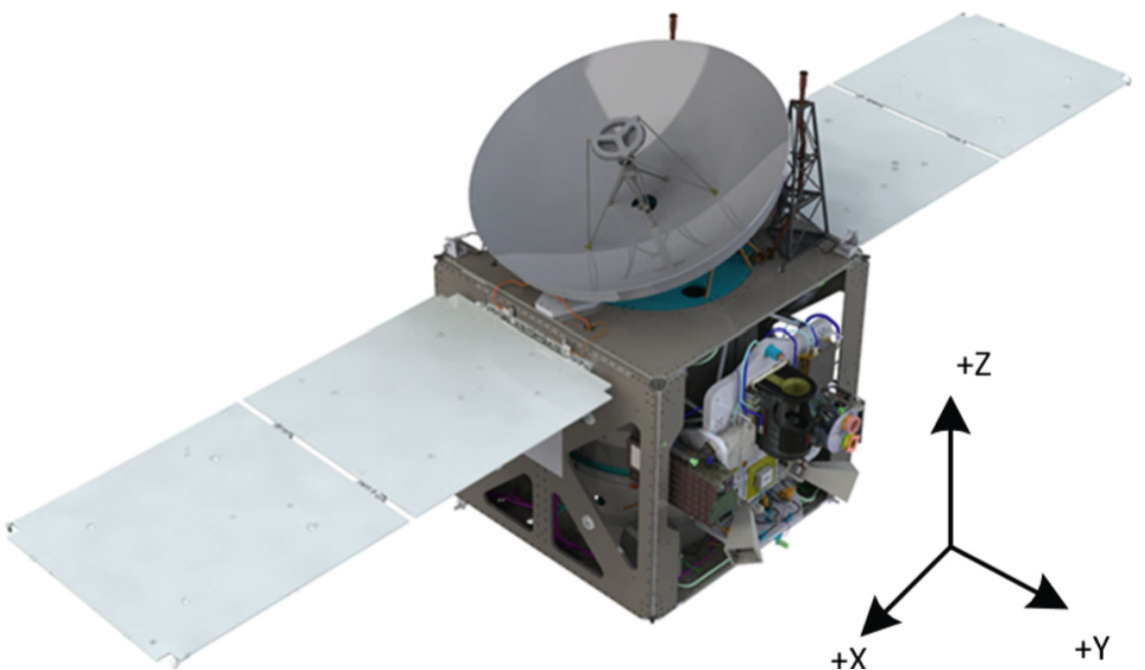
EMM spacecraft without thermal blankets

The spacecraft design is highly tunable for multiple mission types from deep space to Earth orbiting. The architecture can accommodate designs from single string to fully cross-strapped, with varying degrees of radiation robustness. Power system architectures for both high-power (radars, electric propulsion) and low power (imagers, spectrometers) have been developed and are accommodated by the existing hardware. The as-built spacecraft is sized to accept a variety of smaller and larger fuel tanks for different mission designs from GEO station-keeping to planetary injection to small-body rendezvous. Figure 14 shows a CAD rendering of the spacecraft as implemented for EMM.

##### Attitude Determination and Control System

Multiple pointing and safing states provide both fine and coarse attitude determination and pointing control utilizing asymmetric redundancy on both the hardware and software sides. This design effectively enables Sun pointing, Earth pointing, and inertial pointing with high precision (star trackers and reaction wheels), low precision (coarse sun sensors and attitude thrusters), and highly disturbance-resistant (Inertial Measurement Units in combination with both translational and attitude thrusters) pointing states. Science collection (stare, scan, or rotate), battery charging (normal ops or safe), thruster maneuvers (fine momentum dumping or large translational burns), and antenna pointing (precision high gain or coarse low gain) are all accommodated. Algorithm architecture is table driven for easy tuning during both development and flight and can accommodate multiple hardware implementations from larger/smaller thrusters, electric propulsion, higher-precision Inertial Measurement Units (IMUs), or larger/smaller reaction wheels.

##### Electrical Power Subsystem

The simple and robust Direct Energy Transfer architecture consists of four solar panels arranged in two wings with 12 segments consisting of four or five strings per segment, producing over 600 Watts end-of-life at Mars aphelion. These feed a single 78 Amp-hour, 8s52p, Lithium-ion battery. Battery charging and switch control is provided by an internally redundant Power Control Unit (PCU) that consists of slices on an expandable backplane to accommodate (with demonstrated, in-flight heritage) low- or high-power payloads. The current array design accommodates additional panels, as needed. The Field-Programmable Gate Array (FPGA)-based Unit Manager provides redundancy arbitration for both fully- and selectively-redundant C&DH and Power Control architectures, and is the last line of defense in the spacecraft reset architecture. The PCU also supports brown-out recovery.

##### Command and Data Handling (C&DH)

A custom-built C&DH subsystem consists of a Single Board Computer with a LEON processor and RTAX 2000 FPGA, and multiple radiation-tolerant memory locations; a Solid State Recorder with an 8051 processor, an RTAX 4000 FPGA, and 16 GB of science and engineering data storage; two I/O cards (analog and digital interfaces); and a high-heritage low voltage power supply. The current chassis has room for additional interface cards, custom mission-specific cards, or expanded storage capability. The C&DH architecture supports a fully redundant architecture and was designed to utilize an Emergency Mode Controller (EMC asymmetric keep-alive functionality), if required. The as-built EMM avionics architecture utilizes a selectively redundant design without an EMC.

##### Flight Software

The LEON-targeted flight software is based on the NASA open-source cFE/cFS platform running on an RTEMS real-time operating system. Mission-specific applications provide access to Spacecraft subsystems/hardware and are table-driven for configurability. Sequencing capability supports absolute and relative time-tagged command execution and conditional branching. An on-board file system allows file/memory uploads and dumps. The 8051-targeted flight software primarily supports hardware-mediated data ingestion and playback and manages the read and write pointers on the solid state recorder.

##### Telecom

The Frontier Radio from the Johns Hopkins University Applied Physics Laboratory (JHU/APL) combined with a TWTA, a 1.85 meter diameter high gain antenna, and three low gain antennas, plus waveguide, coax and waveguide switches, diplexers, and filters, forms the configurable and highly reliable telecommunication relay utilizing X-band Channel 5 (and left hand circular polarization) on the NASA Deep Space Network for both downlink and uplink. Supporting data rates from 40 bits down / 7.8125 bits up to over a Megabit/sec downlink and 2 kbits up, the EMM telecom system is downlink-bandwidth-limited (by regulation) to approximately 242 kbits/sec with Turbo encoding and BPSK modulation.

##### Propulsion

A pressure-regulated, monopropellant hydrazine system provides both translational and rotational (3-axis, pure couples) impulse. The spacecraft structure is designed to accommodate a range of propellant tank sizes and the Hope Probe utilizes an approximately 800 kg capacity tank operating at approximately 260 psi during flight, and pressure-fed by a 4,000 psi Helium COPV tank. The six, aft-mounted Delta V thrusters produce a combined thrust of 678 Newtons while the eight nominal 0.9-Newton RCS thrusters handle rate damping off the launch vehicle, 3-axis control in Safe Mode, and rotational control during Delta-V (translational) burns. Multiple thruster sizes and configurations are accommodated by the architecture.

##### Thermal

Redundant, active temperature control is provided by both flight-software-controlled and thermostatically-controlled heater circuits. Passive thermal control is provided by radiators and multi-layer insulation. Heater power varies from approximately 10-60% of total Observatory power over the course of the mission.

##### Fault Protection

The spacecraft Fault Protection system is designed to keep the spacecraft power positive, communicative, and thermally stable at all times. This is accomplished via multiple layers of protection and a philosophy of “keep the spacecraft safe” as opposed to “keep the spacecraft (scientifically) operational”. This approach allows a more robust architecture that relies on ground intervention to return the spacecraft to service after a fault, with specific exceptions made for operations during critical events such as MOI. The overall spacecraft architecture was designed around a centralized fault protection engine with key safety features distributed across various sub-systems and processors to provide asymmetric fault detection and responses. Key to the design was the ability to test every aspect of the fault protection system on the ground in keeping with the program’s robust testing philosophy.

#### Performance

In-flight performance of the spacecraft has been nominal across the board with only minor deviations from expected behavior. Spacecraft pointing and stability is better than required, telecom is operating with positive margins in all uplink and downlink configurations, and thermal predicts match expectations with heater zones cycling as expected. The power bus is drawing slightly less current than expected and battery fade is lower than expected with array output slightly outperforming the conservative pre-launch analysis. Data storage and file upload and downloads are performing nominally and system resilience to space weather events has been as designed. The fault protection system has proven to be easy to operate and responds as expected. Safe Mode entry and exit have been successfully demonstrated with the system performing as expected. Trajectory correction maneuvers have been performed and Mars targeting is nominal. Multiple maneuvers have been completed to characterize subsystem performance over and above requirements validation. This additional data provides deeper insight into overall spacecraft performance and will allow the team to fully characterize the expected spacecraft behavior prior to arriving at Mars to begin science operations.

#### Summary

The Hope Probe successfully completed its commissioning activities during the Cruise Phase, and had a successful Mars Orbit Insertion on February 9, 2021. Hope is fully functional, and performing nominally. The spacecraft is successfully supporting all three instruments during the Science Phase.

### Accommodation

Instrument accommodation on Hope was planned with the goals of simplifying interfaces using standardization, reducing schedule dependencies through parallel activities enabled by high fidelity simulators, and ensuring instrument safety throughout flight operations.

**Fig. 15:**
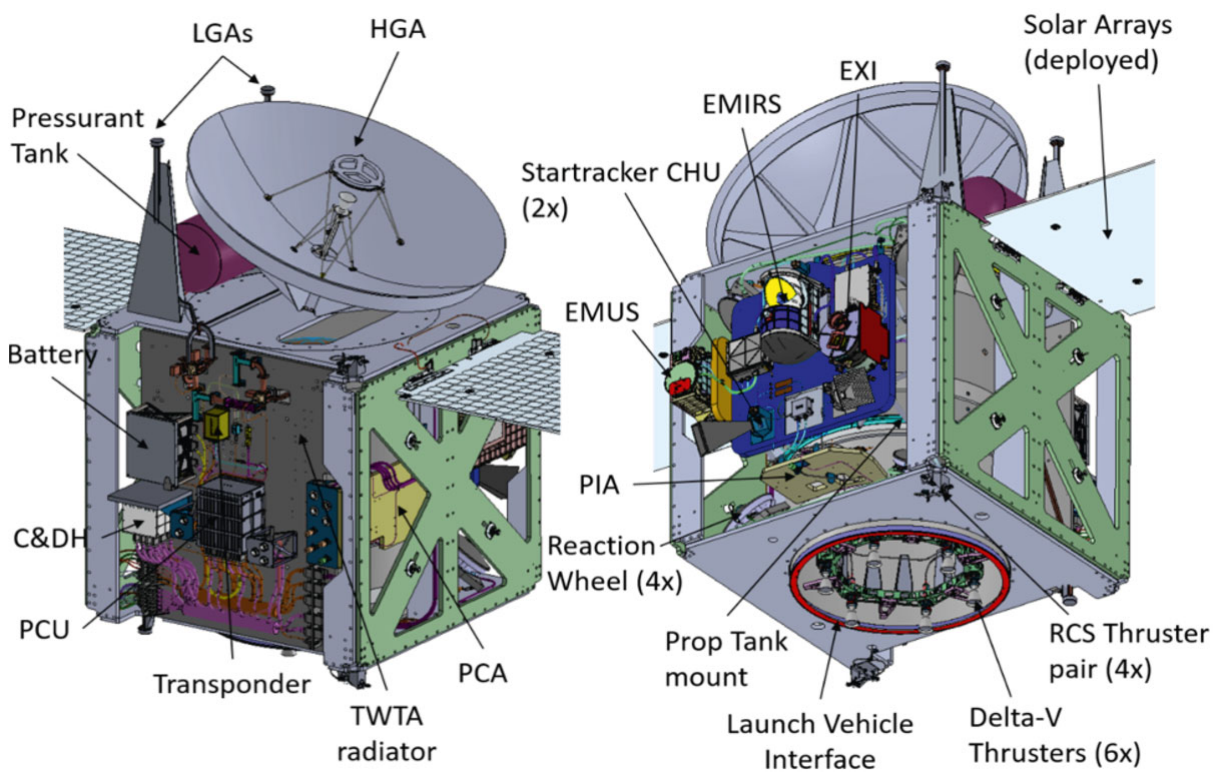
CAD drawing of EMM spacecraft showing the locations of important components, including the science instruments EXI, EMIRS, and EMUS

The EXI, EMIRS, and EMUS instruments were co-located with each other (Fig. 15) to facilitate co-observations of the Mars atmosphere and to simplify spacecraft maneuvering for observational scenarios during science operations. The instruments were attached to a removable instrument panel (IP) constructed of carbon fiber facesheets and thick honeycomb designed to enhance thermal and pointing stability. The instrument locations on the IP simplified instrument purge access and instrument field of view accommodations.

Having the instruments mounted to an independently removable panel increased flexibility of the integration and test activities during observatory integration. The IP was removed during harness routing, purge routing, and thermal blanket installation. The order of integration of the instruments was not constrained by their location on the IP. The star trackers, MIRUs, DPU, and master reference cube were mounted to the IP as well, enabling loose alignment tolerances that didn’t require positional shimming of the ADC sensors and instruments. Location requirements were budgeted and measured in reference to a master reference cube (MRC). Initial alignment surveys were made with the panel off the spacecraft and final surveys were made during environmental testing with the IP installed onto the spacecraft. Jitter analysis, and later in-situ testing, was performed to ensure instrument pointing isolation from reaction wheel activities.

IP thermal control was passive and isolated from individual instrument thermal zones with the exception of survival heater circuits. Electrical interfaces to the instruments were standardized using the same connector configuration for the power connector. The spacecraft flight software (FSW) interface was standardized for all instruments and included a time message, status message, and CCSDS packets.

The instrument teams used a commonly designed electrical spacecraft simulator (SC Sim) provided by the mission to power and command their instrument during instrument level integration at test. The use of the SC sim reduced the technical risk of instrument development, but also reduced Observatory Assembly, Integration, and Test (AIT) schedule risk since the instruments were already testing the interface itself prior to flight delivery to the observatory. Instrument mass models were provided to, and used by, the AIT team to perform mechanical interface verification and spacecraft structural verification testing prior to instrument delivery. Instrument electrical simulators were provided for system testing efforts to verify power and data interfaces. The first instance of this testing was on the spacecraft FlatSat, used to test the interface prior to finalizing instrument FPGA code and board design. When flight instrument deliveries were delayed, integration activities continued on the observatory, in some cases continued into environmental testing, using engineering models with flight equivalent mechanical, electrical, and data interfaces.

All three instruments were purged throughout observatory AIT and up until T-18 hours prior to launch, with limited time off purge of up to 2 hours at a time, in order to reduce accumulation of particulate and non-volatile residue on detector and optical surfaces.

All EMM instruments are sensitive to, and can potentially be permanently damaged by, direct sun down the boresight of the instrument. As such, sun avoidance for instrument safety was developed with this order of accommodation: 1) planning of observational activities to avoid instrument boresight illumination, 2) instruments safe themselves when detecting a sun incursion, typically in the form of closing a door to safe optics or detectors, and 3) the spacecraft initiated a fault response to instrument mechanism position anomaly bits or sun incursion bits. All spacecraft maneuvers planned during science operations were planned with a no observation zone (NOZ) constraint, which was a spacecraft maneuvering keep-out zone cone for the observing instrument(s) during a particular observation scenario.

The instrument science data rate and volume drove the design of the spacecraft solid state recorder (SSR) storage size and read/write speed. Similarly, instrument science data volume was the design driver for the downlink budget.

All instrument to spacecraft interface details were captured in a detailed interface control document started at mission PDR and maintained past launch, with specific implementations captured in the instrument User Guides delivered to the flight operations team.

Overall, the accommodation of science instruments on the Hope spacecraft was straightforward, simple, minimized schedule dependencies during AIT, and minimized spacecraft automated fault responses.

### Planetary Protection

Beginning in 2015, EMM reach out to the international planetary protection community, including COSPAR, NASA, ESA, JAXA, and consultants, to ensure compliance with international policies and community acceptance of the EMM planetary protection program. An early development trade study resulted in the decision for EMM to pursue compliance with probability of Mars impact requirements, as opposed to implementing a bio-burden program which program leadership viewed as a higher risk to development. This decision results from the fact that EMM’s orbit is very large. The planetary protection plan is to therefore demonstrate a very low probability that the spacecraft will ever encounter Mars’ surface or lower atmosphere during the mission. The EMM team has prepared methods to demonstrate that the launch vehicle targets support a <0.01% probability of impacting Mars within 50 years; any part of Hope has a <1% probability or less of impacting Mars within 20 years; and any part of Hope has a <5% probability or less of impacting Mars within 50 years.

The chosen probabilistic approach resulted in trajectory biasing, necessitating rigorous trajectory analyses as well as spacecraft design accommodations to shield pressure vessels from possible micrometeorite impacts. The need for a spacecraft reliability analysis/number was eliminated by adopting a conservative assumption that at each key juncture (launch, TCM-1, TCM-2, MOI, etc.) the spacecraft fails to further execute any maneuver. In other words, the failure probability is assumed to be 100% at each key juncture for the purpose of planetary protection.

## Mission Development and Testing

### Development Timeline

**Fig. 16:**
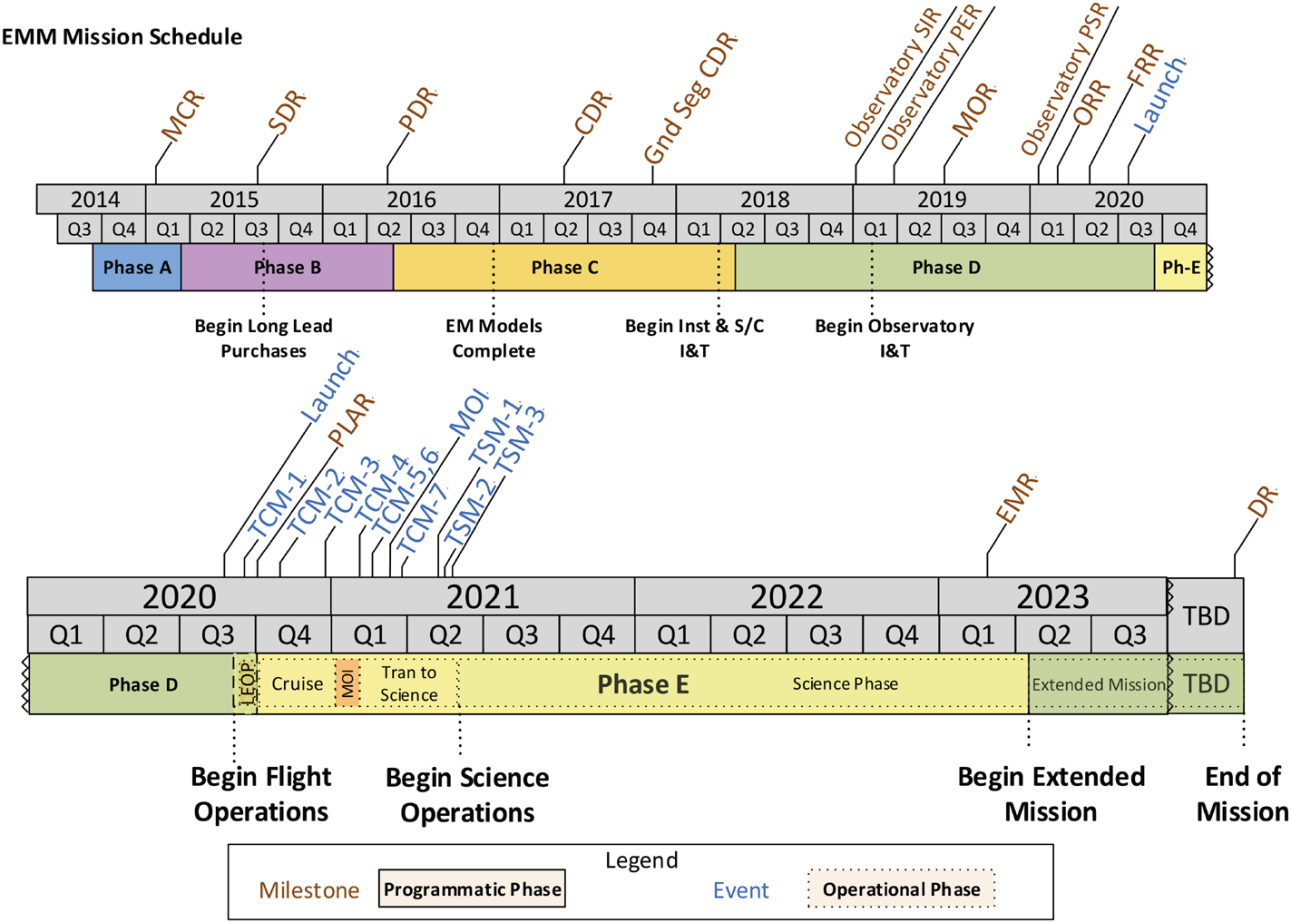
Timeline showing EMM mission development phases (top) and the operational phase of the mission (bottom)

The EMM development timeline was driven primarily by a mission requirement to launch in the summer 2020 Mars launch window, for a 2021 arrival at Mars. A robust and rigid milestone and peer review schedule was established in order to meet the planned launch date of 14 July, 2020 (UTC). The mission concept was formulated during a 100-day study in 2014. The development then employed traditional mission phases common to most spacecraft missions, as shown in Fig. 16, with major reviews at key decision points, and approximately 98 additional reviews of mission systems and subsystems from inception of the mission to launch.

### Assembly, Integration, and Test

The EMM Assembly, Integration, and Test (AIT) program was designed to deliver a fully verified and validated system to orbit. As the EMM observatory is a new design, the test program places special emphasis on hardware safety during integration and system characterization during development to reduce risk to overall mission success. Since one of the primary project goals is the training of Emirati engineers in the design, development, and test of interplanetary spacecraft much of the development of the EMM AIT program was performed from the ground up; this includes the scope and sequence of the test program, design for test requirements, non-flight hardware development, and execution processes. This development was conducted jointly between CU-LASP and partners in the UAE.

#### Assembly, Integration, and Test Program Constraints, Inputs, and Development

The AIT program operated under a set of unique project constraints, which necessitated development of a number of unique solutions.

First, and most importantly, by design the EMM AIT program was unencumbered by organizational processes from previous spacecraft test programs but constrained by personnel and funding availability. As a result, efficiency in requirement identification and selection was required. Individual lessons learned from previous testing experience were leveraged in addition to industrial best-practices where the team assessed them as applicable. Broadly, the AIT program took inputs from both US DoD and Civil government spacecraft testing standards but primarily referenced GSFC-STD-7000A, General Environmental Verification Standard, for development of the test program scope and objectives. Standards such as this were used as reference for the basis for some EMM-specific requirements but were not broadly applied. Significant effort was placed early in development (prior to SRR) to define the smallest possible set of requirements for program success while not constraining those which were deemed necessary.

Like many planetary missions, schedule constraints were the norm. This, coupled with financial constraints, ultimately resulted in a proto-qualification test program selection, which uses the flight article for all test phases rather than using a separate dedicated qualification article. The resulting risk was assessed and accepted by the project and mitigation design strategies implemented. The available resources were applied throughout the AIT program to ensure that both low-level system characterization data was collected and system level performance margin were established.

Finally, due to personnel availability constraints during development on the AIT team, an effort was specifically made to increase efficiency throughout the execution phase. This included efforts to drive commonality throughout all Ground Support Equipment and leverage existing designs wherever possible, procure time-saving hardware handling equipment, drive commonality of interfaces in the flight design where possible, develop standard AIT execution processes, and implement common test software throughout the program.

#### Assembly, Integration, and Test Program Execution

**Fig. 17:**
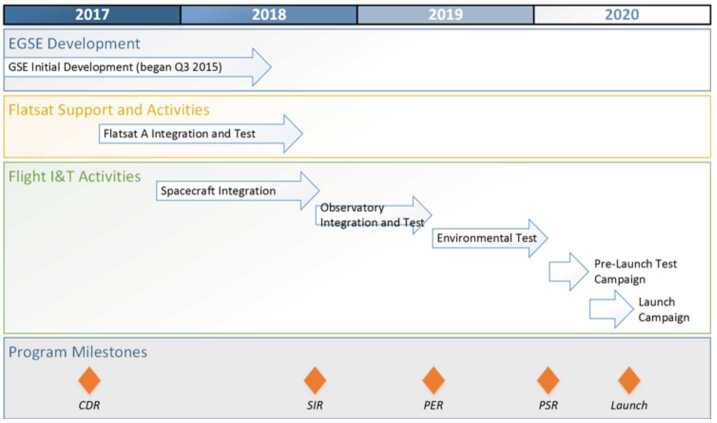
EMM Assembly, Integration, and Test Timeline

The EMM AIT program successfully executed all planned activities within the allocated schedule, arriving at the launch site with slightly more days of schedule margin than originally baselined. The execution timeline is shown in Fig. 17.

#### Spacecraft Integration Plan

**Fig. 18:**
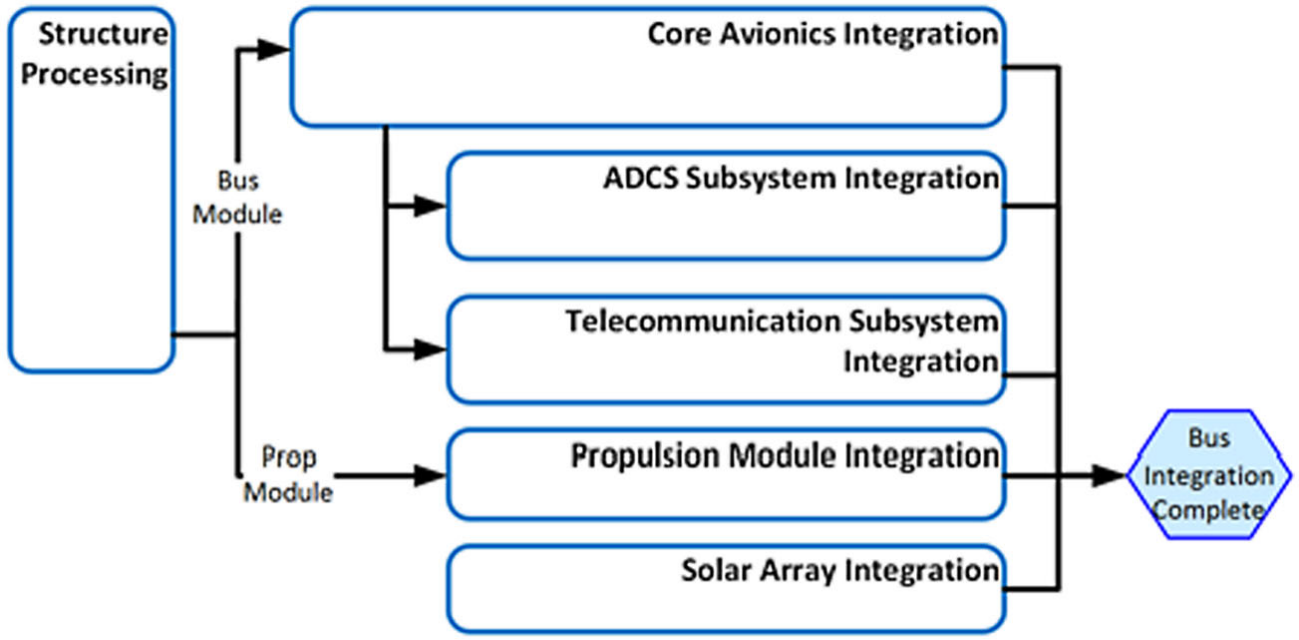
EMM spacecraft integration plan summary

The Spacecraft integration and test plan is designed for maximum flexibility, making use of fundamentally required mechanical, data, and power interfaces as early as possible. An overview of the integration plan is shown in Fig. 18. This allowed the test program to accommodate shifting component delivery schedules without impacting the overall spacecraft integration schedule. This included taking advantage of fundamental spacecraft design features, such as separable spacecraft bus structure from propulsion structure, which allowed the integration schedule to take advantage of available flight hardware.

#### Mission Testing

Testing of the completed EMM Observatory, which includes the spacecraft and instrument payload suite, at the mission level consisted of both interface verifications as well as demonstration of robustness of the EMM design during expected mission scenarios.

**Fig. 19:**
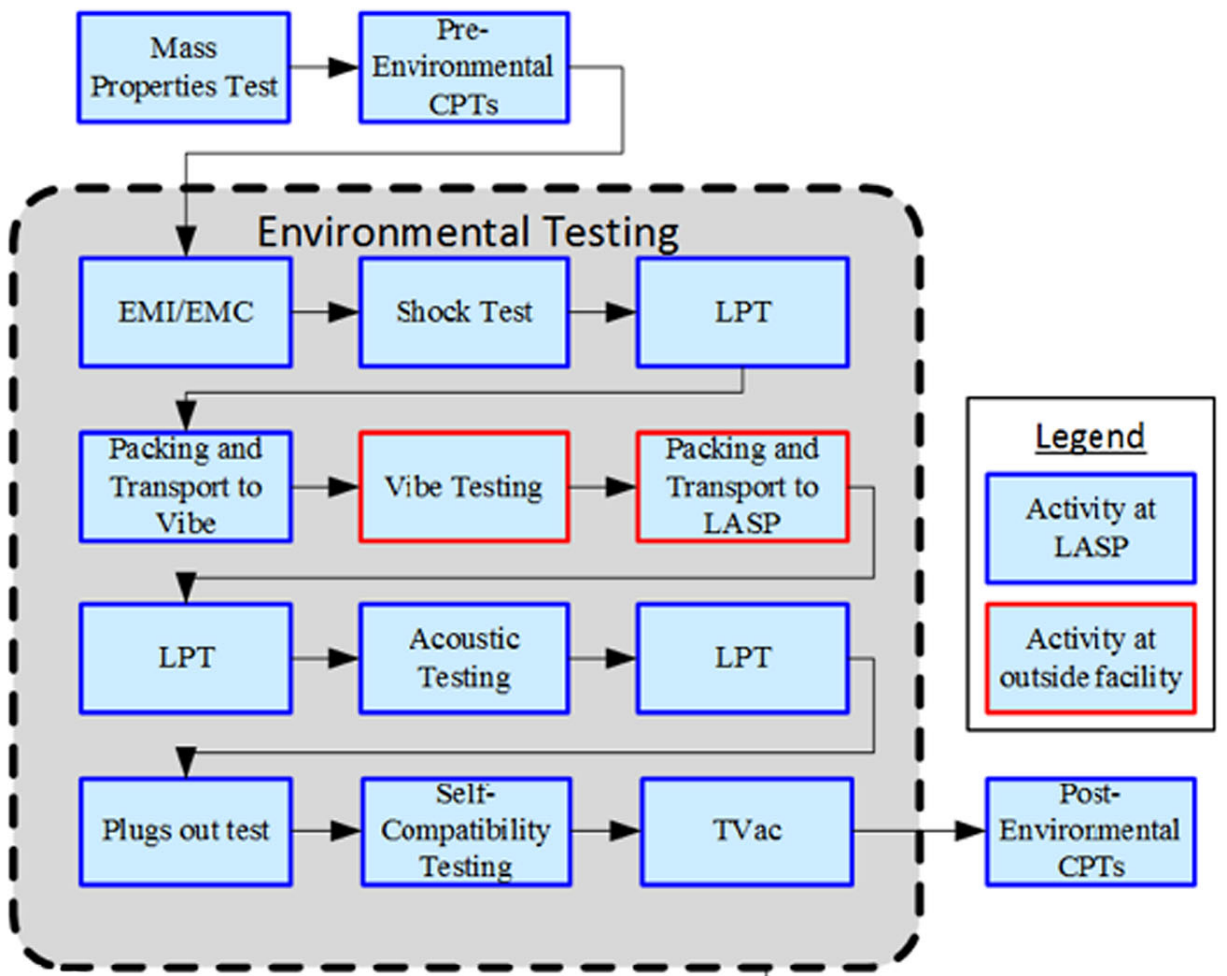
EMM spacecraft integration plan summary

**Fig. 20:**
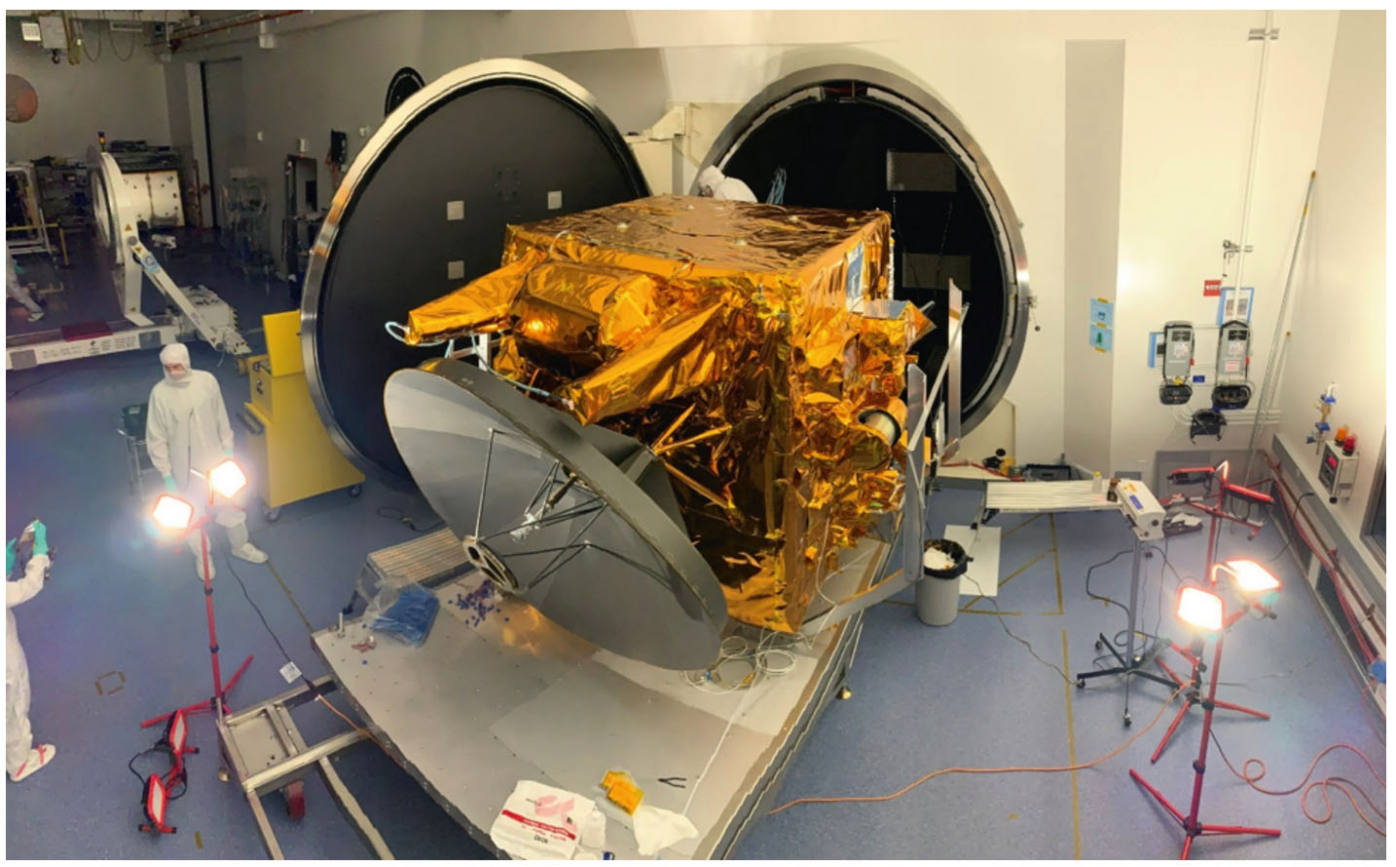
Preparation of the EMM observatory for thermal vacuum testing

In order to reduce both schedule and technical risk the EMM space to ground interface with the NASA Deep Space Network (DSN) was demonstrated early in the program, just after final spacecraft integration was completed. The remaining interfaces with the launch vehicle and the subsequent environmental tests were performed after Observatory integration was concluded. The EMM observatory environmental test program consisted of electromagnetics, launch vibration and acoustics, shock, and thermal testing. The test program design minimized schedule risk by bringing as many of these test environments to the Observatory rather than bringing the Observatory to the test house. This allowed the program to retain technical flexibility and schedule resiliency throughout much of the environmental test program (Fig. 19-20).

The EMM Mission Scenario Tests (MSTs) are designed to demonstrate space segment-level performance of flight scenarios in a test configuration which simulates the in flight configuration as much as possible. For EMM these tests not only demonstrated execution of these scenarios under nominal conditions but also established performance margin by executing these scenarios in the presence of unexpected in-flight anomalies. These anomalies consisted of failing sensors, actuators, and simulated software faults. This demonstrated the resilience of the EMM Observatory design under the unexpected conditions of flight and built confidence for operations.

#### Testing in Dubai

When the environmental testing was completed, the Spacecraft was shipped to the UAE to be further tested with the Mission Operations Centre (MOC). The testing was aimed at making sure that the spacecraft and the MOC can communicate with each other as intended. This included a series of flight-like tests including a Mission Operations Long Duration (MOLD) Test, which simulates a week in the life of the Observatory in flight. This test also helped the team, including the mission operations team, to practice operational procedures on the Observatory as a flight-ready article and not as a test article.

**Fig. 21:**
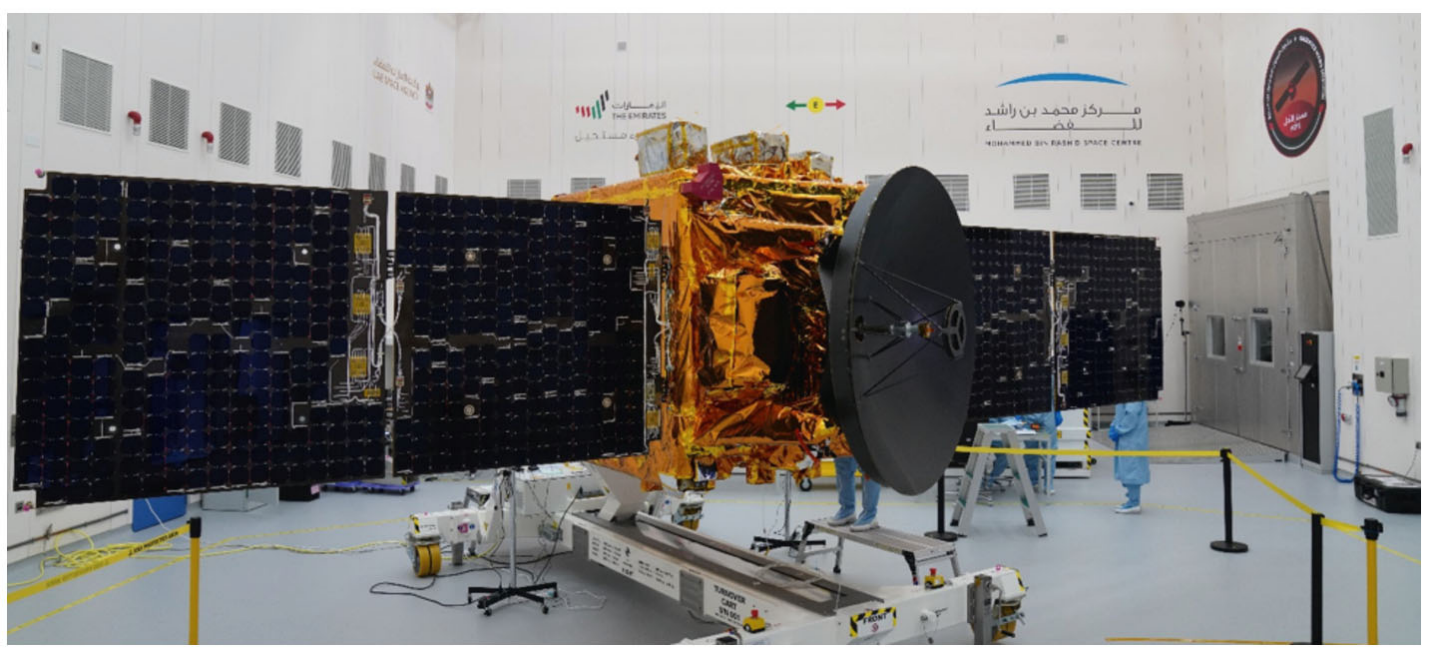
EMM observatory solar array deployment testing

In addition, the Solar Arrays were deployed and re-stowed in Dubai to verify that after being subjected to extreme environments in the environmental test campaign, the Solar Arrays will function as expected (Fig. 21). The pointing alignment of the instruments and the Star Trackers were also surveyed at the MBRSC to assure that the transportation events don’t cause a drastic change to these sensitive components.

Finally, the activities at the MBRSC included an update to the flight software configuration parameters with the most updated flight products and subsequent testing to verify that the spacecraft software package functions as required. After the testing activities at the MBRSC was completed, the spacecraft and Ground Support Equipment were shipped to the Launch Site at Tanegashima, Japan to start the launch campaign.

#### COVID Response

The EMM observatory arrived in Dubai on 9 February, 2020, with the intention of completing a test campaign that included the MOLD test described in Sect. 7.2.5 through the planned departure for the launch site in Japan. However, the rapid onset of the COVID-19 pandemic complicated both the observatory testing in Dubai and the transport of Hope to the launch site in Japan. On 11 March, 2020, the World Health Organization (WHO) declared COVID-19 a pandemic, and several days later the UAE government closed its air space, restricting all flights into the country. In late March, 2020, the Dubai government instituted a mandatory stay at home order for anyone without government authorization to work; the EMM team quickly obtained such an order. In early April, 2020, the Japanese government restricted entry into the country from the UAE and the United States. As countries across the world began to clamp down on international travel, and then to travel outside of the home, the EMM management team assessed tightening constraints in the three countries three times daily.

A number of changes in the testing and delivery schedule and logistics were required to ensure that EMM could arrive at the launch site in Japan in time for its launch window. The test campaign in the UAE was accelerated and the observatory was configured for transport as soon as possible, which took approximately three weeks. The team in MBRSC worked with the UAE and Japanese governments to approve a team of Emirates and a single American to enter Japan via a chartered flight weeks in advance of the planned shipment of the observatory in order to quarantine for two weeks. This team (from MBRSC, the UAE Space Agency, and LASP) was essential for offloading the Observatory from the aircraft and transporting it to the island of Tanegashima. The team remained in Japan for the entirety of the launch campaign. Waivers from the governments of the UAE, Japan, and Russia had to be obtained to land the Volga-Dnepr Airlines (VDA) Antonov AN-124 aircraft in the UAE for loading of the observatory and immediate departure to Japan. MBRSC in coordination with LASP management arranged for a separate small team from LASP to travel to the UAE and quarantine for two weeks to assist the MBRSC team with the final reconfiguration for air transportation. A small cohort of EMM team members rode on the aircraft to Japan to assist with offloading and launch site activities. MBRSC and UAE government coordinated with the Japanese government to expedite and provide the approval for another small LASP team to travel to Japan to support launch site operations.

All hurdles associated with COVID-19 were successfully navigated and the mission launched on time. In all, EMM team members spent 420 days (more than 10,000 hours) in quarantine to support the UAE test campaign and the Japan launch campaign.

## Operations and Data Availability

**Fig. 22:**
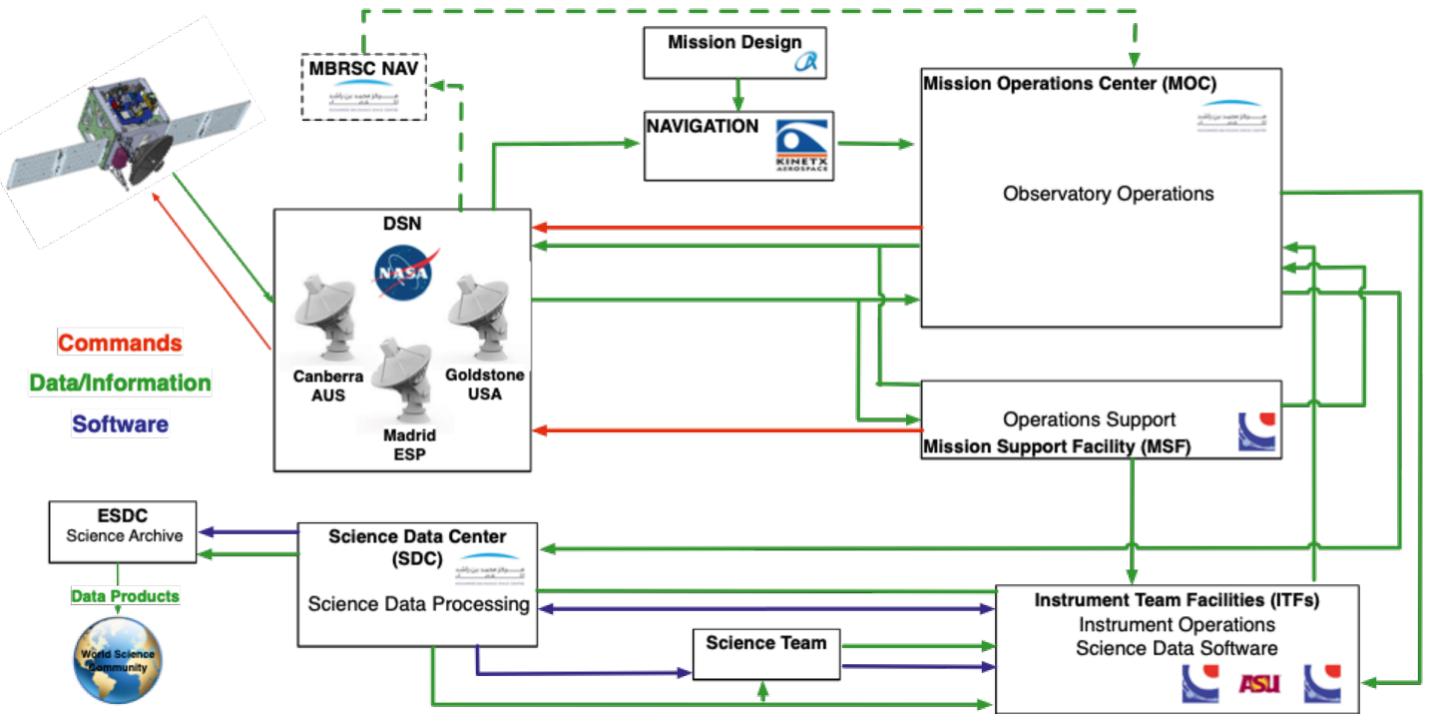
EMM Ground Segment Operations Data Flows

The EMM Ground Segment (GS) is distributed world-wide with elements and associated facilities located on four different continents. Each of the elements within the ground segment make important contributions to the success of EMM. MBRSC in the UAE hosts two key elements within the ground segment: the Mission Operations Center (MOC) and the Science Data Center (SDC). The MOC is the primary operations facility and coordinates activities with the Mission Support Facility (MSF) at LASP as well as the other members of the ground segment. The SDC is a partnership between the MBRSC and LASP and is supported by the Instrument Team Facilities (ITFs) at LASP and Arizona State university. Command uplink, telemetry downlink and tracking services are provided by NASA’s Deep Space Network with antennas located in Canberra Australia, Madrid Spain, and Goldstone USA. The Flight Dynamics System (FDS) is supported by KinetX Aerospace, Advanced Space, LASP and the MBRSC. The operational data flows between the EMM elements can be seen in Fig. 22.

The EMM Ground System elements: MOC, MSF, ITFs, SDC, Navigation, and the DSN work together with other mission elements to plan and execute the Observatory operations, analyze the data for health and performance assessment, and process and distribute the science data over the lifetime of the mission.

### Mission Operations

**Fig. 23:**
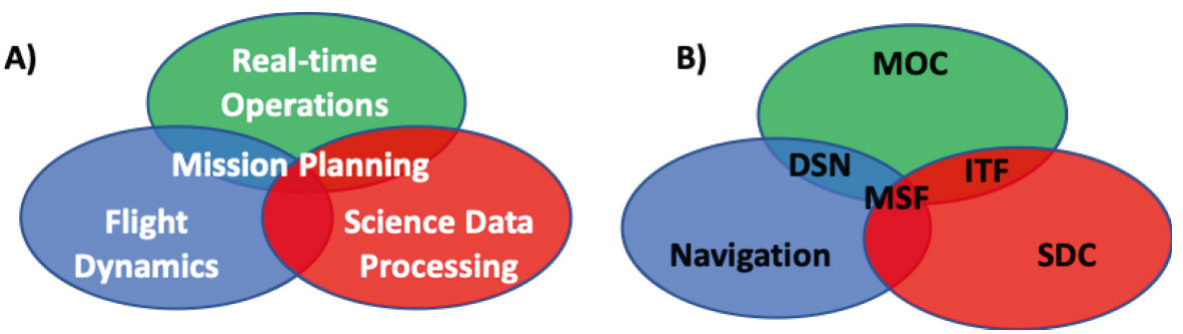
**A**) Relationship of mission operations functions. **B**) Elements supporting flight operations

The Ground Segment supports four key functions for EMM: Real-time operations, Flight Dynamics, Science Data Processing, and Mission planning. The relationship of these functions and the elements which support them can be seen in Fig. 23.

The MOC leads the real-time operations function for EMM. It consists of the hardware and software systems used to command, control, and receive telemetry to be used in performing scientific observations and assessing the health and safety of the EMM Observatory. The Operations Team in the MOC is responsible for planning, creating, reviewing, uploading and approving the command sequences to be used onboard EMM. The MOC also receives and processes the downlinked data from the Observatory and distributes the science and instrument housekeeping data to the Science Data Center.

The MSF consists of similar hardware and software systems as in the MOC and can also be used to command, control, and receive telemetry from the EMM Observatory. Trained staff at the MSF can support flight operations as necessary. The global distribution between the MOC and MSF gives the EMM Operations Team a real advantage for maximizing facility redundancy and staffing capacity.

The Flight dynamics function is supported by the Navigation, Mission Design, DSN, MOC and MSF teams. The Navigation element is responsible for computing the EMM spacecraft reconstructed trajectory in flight using radiometric tracking data from the DSN and spacecraft data from the MOC. Navigation is also responsible for generating the spacecraft predicted trajectory and for designing spacecraft maneuvers. Mission Design is responsible for defining the overall EMM mission trajectory (the “reference trajectory”) from launch through science operations, such that the reference trajectory meets the EMM science and mission requirements. The MBRSC Navigation (M-Nav) component is a technology demonstration effort within the flight dynamics function. It is developing and validating tools to create trajectory predictions and reconstructions for future missions.

The ITF for each instrument is responsible for building and maintaining a repository of engineering information supporting the instrument, instrument operations, and science data production and support. ITF work includes providing instrument-planning requests, reviewing operational plans, uplink products, and reporting instrument health and safety. The Instrument Teams provide instrument monitoring and state management, provide operational command requests, and support instrument calibration. Furthermore, the Instrument Teams will work with SDC to set up their L1 and L2 processing software on the SDC and create L3 datasets for delivery to the SDC.

The Mission planning function requires support from each element within the ground segment. It receives inputs from all components of the mission. It adjudicates, prioritizes, and checks constraints on each of the requests. The end result of the mission planning process is a set of validated command sequences ready for execution on the observatory.

### Science Data Center

The Science Data Center (SDC) is responsible for handling the mission’s science data, and acts as a central hub where both the EMM team and the scientific community can retrieve the data. The SDC primarily serves 3 distinct roles: science data processing, data management, and data access.

#### SDC Implementation

All core SDC functionality is implemented using AWS’s infrastructure and services. Physically, the infrastructure is in the AWS’s Ireland region, which was chosen as a rough half-way point between the United States and the UAE. All science data files and all files relevant for using the science data are stored in AWS S3 buckets, which provide 99.999999999% data durability. The majority of data processing takes advantage of Amazon’s Batch processing service, and data is disseminated via AWS’s API Gateway tool.

#### Data Processing

The SDC receives Level 0 data, SPICE kernels, and engineering data from the MOC several times per week. As new data arrives, the SDC processes it into Level 1, Level 2, and Quicklook science data files using software developed by the EXI, EMUS, and EMIRS instrument teams. As the algorithms in the software are further refined, the SDC supports reprocessing of the full dataset to newer versions. Generating Level 3 products is the responsibility of the instrument teams and does not occur at the SDC. However, all Level 3 data generated will be delivered to the SDC by the instrument teams, where they are stored and managed alongside the other science files.

#### Data Management

The SDC maintains a secure, stable repository of all science data throughout the mission. A file naming convention is enforced that allows users to determine general information about the file’s contents (e.g. - date range, observation type, version number, etc). This file metadata is indexed in a database managed by the SDC, which other SDC tools and SDC users can then use to query for data. To ensure that data is not lost, weekly backup copies of all data files and databases are archived in a geographically distinct area from the SDC. Additionally, all files will be stored on hardware located in the UAE.

#### Data Access

The SDC provides the mission’s science team direct access to the SDC’s primary storage locations, where they are able to sync all data files to local servers directly. For all others who wish to access the EMM data, the SDC maintains a public website (https://sdc.emiratesmarsmission.ae/) called the Emirates Space Data Center (ESDC), as well as several Application Programming Interfaces (APIs) which serve as the primary mechanism for retrieving the mission’s science data. The ESDC will also contain several interactive tools that allow users to search and download specific data sets, such as a data search page, availability charts, and Quicklook file viewers.

### Data Availability

All science data from EMM will be released to the public for the benefit of the international science community. There are no proprietary periods associated with any of the EMM data products, and the scientific community outside the EMM team will have timely access to the scientifically useful products (Levels 2+) through the Emirates Space Data Center (ESDC). The ESDC will provide multiple data access mechanisms to the science community in order to retrieve science data from the ESDC including the ESDC website and published Restful API. The ESDC website contains detailed information for the science community on how to retrieve data from the ESDC using the available mechanisms.

Assuming all goes well with orbit insertion and science phasing, the first release of Level 2 science data products will take place approximately 4 months after the start of the science phase, on September 1, 2021, and will include data from the first 3 months of observations (including the capture orbit). After the initial release, subsequent releases will take place every three months and contain three months’ worth of data subsequent to the first release. The first release of Level 3 science data products will take place on December 1, 2021, at the same time as the second Level 2 release, and will include data from the first 3 months of observations (including the capture orbit). After the initial release of Level 3 data, subsequent releases will take place every three months and contain three months’ worth of data subsequent to the first release. Level 3 releases may initially contain only partial science retrievals depending on the progress of the Level 3 pipeline; complete Level 3 results will be available by end of project. Level 0 and 1 data will be placed in the ESDC archive at the end of the mission as part of the SDC decommissioning. These products will be available to the public at that time, but not before then, as they are not expected to be useful to the science community during the mission.

## Summary

The Emirates Mars Mission was successfully conceived, designed, and implemented in less than six years. Now in orbit around Mars, it is using three instruments to obtain high quality science measurements of the Martian atmosphere on global scales that will contribute to our understanding of atmospheric variability on daily and seasonal timescales, as well as processes that contribute to the escape of water from the atmosphere. The mission satisfies several important programmatic objectives for the UAE, including objectives related to capacity-development in both the engineering and science sectors. The mission team represents an integrated group of engineers and scientists from three continents, that worked together to ensure a successful launch during a global pandemic. The team will continue to work together to operate the mission, obtain data, and complete science analyses.

## Acronym List

ADC: Attitude Determination and Control
AIT: Assembly, Integration, and Test
API: Application Programming Interface
ASU: Arizona State University
AWS: Amazon Web Services
BOS: Big Observation Sequence
BPSK: Binary Phase Shift Keying
C&DH: Command and Data Handling
CAD: Computer-Aided Design
CCSDS: Consultative Committee for Space Data Systems
ConOps: Concept of Operations
COPV: Composite Overwrapped Pressure Vessel
COSPAR: Committee on Space Research
COVID-19: COrona VIrus Disease 2019
CU: University of Colorado
DLaTGS: Deuterated L-alanine doped Triglycine Sulfate
DPU: Data Processing Unit
DR: Decommissioning Review
DSN: Deep Space Network
Ebox: Electronics Box
EMC: Emergency Mode Controller
EMIRS: Emirates Mars InfraRed Spectrometer
EMUS: Emirates Mars Ultraviolet Spectrometer
EMM: Emirates Mars Mission
EOP: Early Operations Phase
ESA: European Space Agency
ESDC: Emirates Space Data Center
EXI: Emirates eXploration Imager
FDS: Flight Dynamics System
FITS: Flexible Image Transport System
FOV: Field of View
FPGA: Field Programmable Gate Array
FSW: Flight Software
GEO: Geostationary
GS: Ground Segment
HDU: Header/Data Unit
HVPS: High Voltage Power Supply
I/O: Input / Output
IFOV: Instantaneous Field of View
IMU: Inertial Measurement Unit
IP: Instrument Panel
ITF: Instrument Team Facility
JAXA: Japan Aerospace Exploration Agency
JHU/APL: Johns Hopkins University Applied Physics Laboratory
LASP: Laboratory for Atmospheric and Space Physics
LMD-MGCM: Laboratoire de Météorologie Dynamique - Mars General Circulation Model
LO: Low
LPT: Limited Performance Test
L_*S*_: Solar Longitude
LUT: Look-up Table
LV: Launch Vehicle
M-Nav: MBRSC Navigation
MARCI: Mars Color Imager
MBRSC: Mohammed Bin Rashid Space Centre
MCP: Microchannel Plate
MEPAG: Mars Exploration Program Analysis Group
MER: Mars Exploration Rover
Mini-TES: Miniature-Thermal Emission Spectrometer
MOC: Mission Operations Center
MOI: Mars Orbit Insertion
MOLD: Mission Operations Long Duration
MOS: Medium Observation Sequence
MO-TES: Mars Observer Thermal Emission Spectrometer
MGS-TES: Mars Global Surveyor Thermal Emission Spectrometer
MIRU: Micro Inertial Reference Unit
MRC: Master Reference Cube
MRO: Mars Reconnaissance Orbiter
MSF: Mission Support Facility
MSO: Mars-Solar-Orbital
MST: Mission Scenario Test
NASA: National Aeronautics and Space Administration
NOZ: No Nadir Observation Zone
OTES: OSIRIS-REx Thermal Emission Spectrometer
PCU: Power Control Unit
PDR: Preliminary Design Review
PDS: Planetary Data System
PLAR: Post-Launch Assessment Review
PMO: Project Management Office
RCS: Reaction Control System
RTEMS: Real-Time Executive for Multiprocessor Systems
SAG: Science Advisory Group
SC Sim: Spacecraft Simulator
SDC: Science Data Center
SOS: Small Observation Sequence
Sp. SOS: Special Small Observation Sequence
SPICE: Spacecraft, Planet, Instrument, C-matrix, Events
SRR: System Requirements Review
SSL: Space Sciences Laboratory
SSR: Solid State Recorder
STM: Science Traceability Matrix
TCM: Trajectory Correction Maneuver
TSM: Tansition to Science Maneuver
TWTA: Traveling Wave Tube Amplifier
U-OS1: EMUS Type I Observation
UAE: United Arab Emirates
U.S.: United States
UTC: Coordinated Universal Time
UV: Ultraviolet
UVIS: Ultraviolet Imaging Spectrograph
VDA: Volga-Dnepr Airlines
VHI: Very High
VIS: Visible
VLO: Very Low
WHO: World Health Organization
XDL: Cross-Delay Line
